# A perception into binary and ternary copper (II) complexes: synthesis, characterization, DFT modeling, antimicrobial activity, protein binding screen, and amino acid interaction

**DOI:** 10.1186/s13065-023-00962-x

**Published:** 2023-06-14

**Authors:** Doaa S. El-Sayed, Eman M. Tawfik, Amel F. Elhusseiny, Ali El-Dissouky

**Affiliations:** Chemistry Department, Faculty of Science, Alexandria University, 2 Bagdad Street, P.O. Box 2-Moharrem Beck, Alexandria, 21321 Egypt

**Keywords:** Copper(II) complexes, Fluorescent probe, Topological analysis, Computational studies, Molecular docking

## Abstract

Ensuring healthy lives and promoting well-being for all at all ages is the third goal of the sustainable development plan, so it was necessary to identify the most important problems that threaten health in our world. The World Health Organization declared that antibiotic resistance is one of the uppermost global public health threats facing humanity and searching for new antibiotics is slow. This problem can be approached by improving available drugs to combat various bacterial threats. To circumvent bacterial resistance, three copper(II) complexes based on the pefloxacin drug were prepared and characterized using analytical, spectroscopic, and thermal techniques. The resulting data suggested the formation of one octahedral binary and two distorted square pyramidal ternary complexes. Fluorescence spectra results revealed the formation of a turn-on fluorophore for amino acid detection. Computational calculations investigated quantum and reactivity parameters. Molecular electrostatic potential profiles and noncovalent bond interaction-reduced density gradient analysis indicated the active sites on the complex surface. The complexes were subjected to six microbial species, where the octahedral binary complex provoked its antimicrobial potency in comparison with ternary complexes. The enhanced antimicrobial activity against gram-negative bacterium *E-coli* compared to gentamicin was exhibited by the three complexes. Docking simulation was performed based on the crystal structure of *E. coli* and *S. pneumoniae* receptors using 5I2D and 6O15 codes. The binary complex exhibited a potent fitness score with 5I2D (TBE = − 107 kcal/mol) while ternary complexes displayed the highest docked score of fitness with 6O15.

**Supplementary Information:**

The online version contains supplementary material available at 10.1186/s13065-023-00962-x.

## Introduction

The wide use of antibiotics demonstrated the key issue in the rise of multidrug resistance in pathogens, which is why the clinical efficiency of the existing drugs became defenseless [1], causing a variety of illnesses that can end in death. Consequently, this permit scientists to develop novel drugs or modify the existing commercial drugs to enhance their efficiency to combat numerous diseases, which may cause serious problems to humankind [2].

Fluoroquinolones comprise a class of antibiotics that can effectively inhibit the replication of bacterial DNA gyrases [3, 4]. Fluoroquinolones form a ternary complex with the DNA and bacterial enzymes, cleave the bacterial DNA, and encumber bacterial replication [3–5] which in turn treat severe infections [6].

To avoid the problem of misuse of these antibiotics, the complexation of fluoroquinolones with transition and non-transition metal ions was used as an alternative to conventional drugs [7–11]. However, the combinations of metal with pharmaceuticals expand the activity of the drug, reduce their toxicity, improve the ability to act as regulators of gene expression and serve as microbiological tools [12–15]. Fluoroquinolones coordinate with metal ions as bidentate ligands via the groups which are responsible for their antimicrobial activity, one carboxylate and pyridone oxygens [7–11], producing a stable chelated complex [10, 11].

Copper fluoroquinolone complexes proved to be the most stable complexes, copper complexes also presented bactericidal and fungicidal action [16, 17] and displayed high activity against a variety of diseases, and tumors. Besides, fluoroquinolone copper(II) complexes bind strongly to calf-thymus DNA (CT DNA) and human or bovine serum albumin protein with high binding constant values [18].

**Scheme 1:**
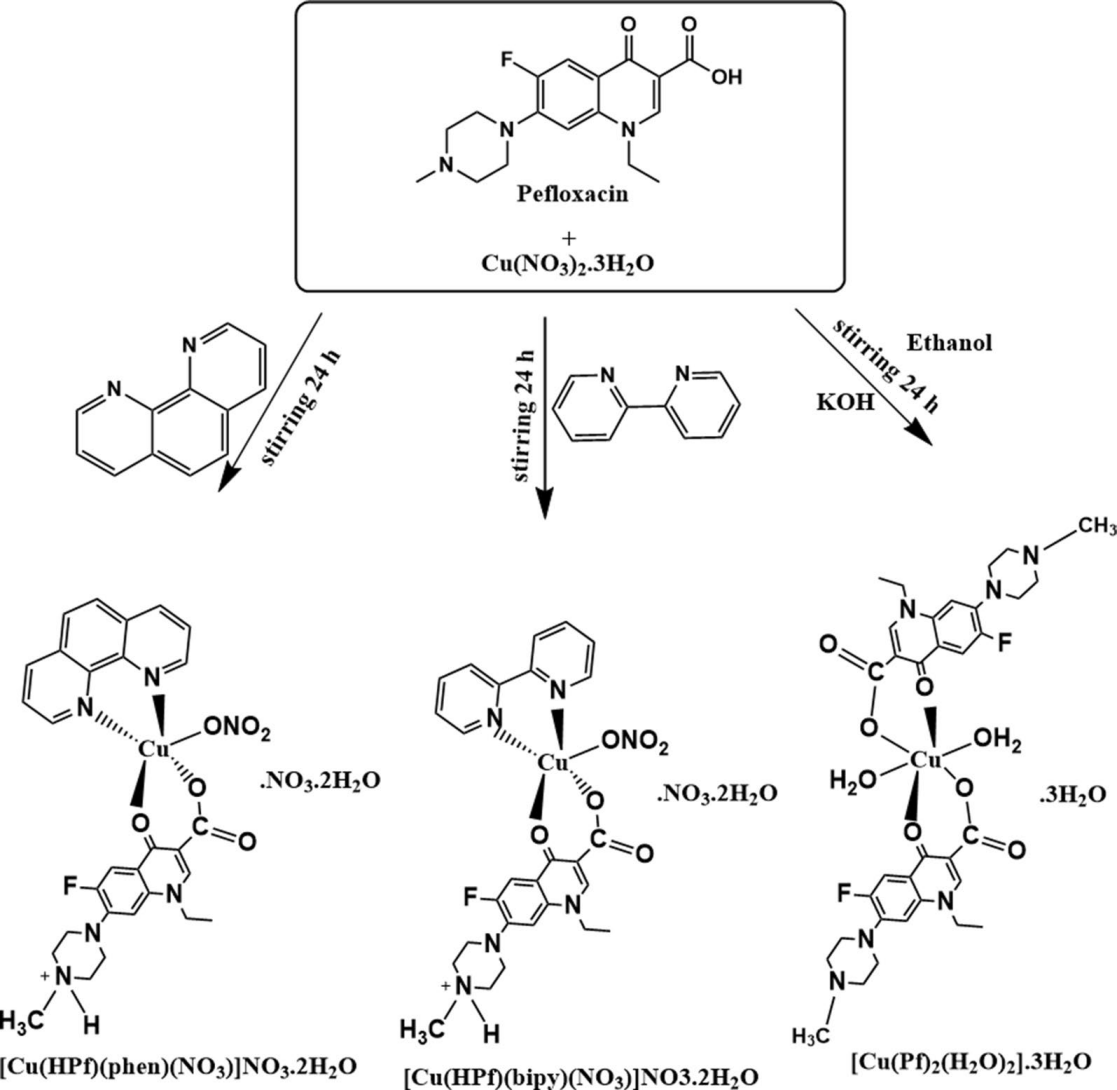
Synthesis of copper(II) based pefloxacin complexes

More active mixed-ligand copper complexes which are prepared from primaryligands as fluoroquinolones and a secondary ligand as heterocyclic ligand containing nitrogen donor, for example, 2,2’-bipyridine and 1,10-phenanthroline, bathophenanthroline, imidazole, and glycine amino acid [19–26]. On this basis, Pefloxacin (HPf), [1-ethyl-6-fluoro-7-(4-methylpiperazin- 1-yl)-4-oxo-quinoline-3- carboxylic acid] (Scheme 1), belongs to the family of antibacterial agents that hinders bacterial DNA replication [27].

Despite pefloxacin had [27], high bioavailability, and a long half-life various bacteria have developed resistance against it and various strategies to enhance its potency were adopted.

Pefloxacin metal complexes displayed higher antimicrobial inhibitory activity than free pefloxacin [27]and revealed a higher intrinsic binding constant to CT-DNA [28, 29].

Mixed ligands metal complexes of pefloxacin-imidazole and pefloxacin and ascorbic acidwere synthesized, characterized, tested against various microorganisms, [27]. To the best of our knowledge, the number of metal pefloxacin complexes reported in various studies is quite limited, specifically, copper pefloxacin mixed ligands using the nitrogen donor heterocyclic ligands 1,10-phenanthroline (phen) and 2,2′-bipyridine (bipy) as secondary ligands, these ligands proved to be more active against microorganisms and showed promising insights against resistant bacterial strains [19–26]. As part of our research area is developing powerful metal-based compounds against antibiotic resistance [17–19], herein we report the synthesis of pefloxacin copper complexes in the absence and presence of 1,10-phenanthroline (phen) and 2,2′-bipyridine (bipy), to study the mode of coordination and the biological activities of the obtained complexes.

The resultant copper(II) complexes were fully characterized using analytical, molar conductance, spectroscopic and thermal techniques.

The fluorescence spectra of pefloxacin were studied both in the presence and absence of copper ions, and the effect of various amino acids on the fluorescence properties of the copper-pefloxacin complex was explored.

In addition, density functional theory (DFT) calculations were performed to estimate some important parameters related to their activity and stability. The type of interaction between the ligand and receptor in molecular docking was examined by natural bond orbital (NBO) analysis. The electrophilic and nucleophilic molecular centers were predicted by molecular electrostatic surface potential (MEP) analysis.The antimicrobial activity was evaluated against bacterial strains, *B. subtilis, S. pneumoniae, E. coli, P. aeruginosa,* and *C. albicans and A. fumigatus* as fungal strains. Drug design and bioinformatics tools were demonstrated to illustrate different interaction types present between the docked compound and protein. *E. coli* and *S. pneumoniae* target using 5I2D and 6O15 codes were taken as receptors for the bio-ligand and its copper complexes.

## Experimental

### Materials and methods

Pefloxacin mesylate dihydrate (HPf), 1,10-phenanthroline (phen), and 2,2′-bipyridine (bipy) were purchased from Sigma–Aldrich Chemical Co. Alanine, proline, aspartic acid, copper nitrate trihydrate, potassium hydroxide, potassium dihydrogen orthophosphate, and sodium hydroxide were used as received from Merck Chemical Company. Nitric acid (68%), Hydrochloric acid, dimethyl sulfoxide (DMSO, Aldrich), acetone, diethyl ether, and ethanol (BDH-PROLABO), were of analytical grade.

### Physical measurements

Carbon, hydrogen, and nitrogen analysis were recorded using an elemental analyzer Perkin-Elmer 240B. Complexometric titration was carried out to determine the copper content [30]. An electrothermal melting point apparatus detected the melting points. HI 8033 HANNA conductivity meter was used to measure the molar conductivity of a 1.00×10^−3^ M DMSO solution at 25.0 ±1.0 °C. A 500 UV–Vis spectrophotometer recorded the ultraviolet–visible spectra for 1.00×10^-5^–5.00×10^−3^M DMSO solutions at room temperature. A Perkin-Elmer model IS 55 fluorescence spectrometer measured the fluorescence spectra at 25.0 ±1.0 °C with freshly prepared solutions. The pH measurements were performed using a Digital Orion pH/ISE meter. FT-IR spectra were studied in the range of 4000-500 cm^-1^ at 25 °C from KBr pellets of 3 mm thickness, using a Perkin-Elmer FT-IR 1650 Spectrophotometer. The mass spectra were performed by the electron ionization technique at 70 eV using a Thermo Scientific Singing Instrument in Cairo University. Electron paramagnetic resonance (EPR) spectra were studied by an EPR spectrometer (Bruker, EMX) at 25.0 ±1.0 °C, X-band frequency 9.8 GHz, with modulation frequency 100 kHz, standard cylindrical resonator (ER 4119HS) and external standard DPPH. Thermal analyses were carried out on LINSEIS STA PT1000 thermogravimetric analyzer in the temperature range of 25 °C–1000 °C with a maximum weight sample of 10 mg placed in a platinum crucible under a nitrogen atmosphere with a 30 mL/min flow rate and a heating rate of 10 °C/min.

### Synthesis of copper complexes

#### Synthesis of [Cu(Pf)2(H2O)2]0.3H2O

A solution of copper nitrate trihydrate (40 mg; 0.166 mmol) in ethanol (10 mL) was added to a mixture of HPf (151.8 mg; 0.33 mmol) and KOH (19.0 mg; 0.33 mmol) in ethanol (15 mL). The resulting mixture was stirred for approximately 24 h at 25 °C **(**Scheme 1**)**. A blue precipitate was obtained, filtered off, washed with distilled water followed by ethanol and diethyl ether, and dried under vacuum at 60 °C for 24 h.

Yield 63%; m.p. 250 °C; Color Blue. Anal. Calc. for C34H48F2N6 O11Cu, (%): C, 50.08; H, 5.91; N, 10.26; Cu, 7.77. Found (%): C, 49.91; H, 5.74; N, 10.23; Cu, 7.37. Λm (Ω^-1^mol^-1^cm^2^) = 4; FTIR (ν, cm^-1^): (O-H/H2O) 3414, ν(C=O) pyridone 1627, ν(COO^-^)asym1585, ν(COO^-^)sym1382, (M-O) 496. UV–Vis (λmax, nm): 282, 344, 723,967. ESR: g∥= 2.31, g⊥= 2.07; ˂g˃= 2.15; G=4.5; A||=170; ƒ=136; α2=0.85.

#### Synthesis of [Cu(HPf)(bipy)(NO3)]NO3.2H2O

A solution of copper nitrate trihydrate (80 mg; 0.33 mmol) in ethanol (10 ml) was added to a mixture of bipy (52 mg; 0.33 mmol) and pefloxacin (151.8 mg; 0.33 mmol) in ethanol (20 ml). The formed mixture was stirred for approximately 24 h at 25 °C (Scheme 1**)**. A greenish-blue precipitate was obtained, separated out, washed with distilled water, ethanol then diethyl ether, and finally dried under vacuum.

Yield 78%; m.p. 278 °C; Color Greenish blue solid. Anal. Calc. for C27H32FN7 O11Cu (%): C, 45.48; H, 4.52; N, 13. 75; Cu, 8.91. Found (%): C, 45.50; H, 4.50; N, 13.70; Cu, 8.64. Λm (Ω^-1^mol^-1^cm^2^) = 20; FTIR (ν, cm^−1^): (O-H/H2O) 3434, ν(C=O) pyridone 1635, ν(COO^-^)asym 1589, ν(COO^-^)sym 1386, (M-O) 517. UV–Vis (λmax, nm): 283, 310,347,419, 649. ESR: g_∥_=2.27, g_⊥_=2.06; ˂g˃= 2.13; G=4.5.

#### Synthesis of [Cu(HPf)(phen)(NO3)]NO3.2H2O

A solution of copper nitrate trihydrate (80 mg; 0.33 mmol) in ethanol (10 ml) was added to a mixture of phen (66 mg; 0.33 mmol) and pefloxacin (151.8 mg; 0.33 mmol) in ethanol (20 ml). The obtained mixture was stirred for approximately 24 h at 25 °C (Scheme 1). A dark blue precipitate was obtained, separated off, washed with distilled water several times followed by hot ethanol and diethyl ether, and finally dried under vacuum for 24 h.

Yield 80%; m.p. 260 °C; Color Dark blue solid. Anal. Calc. for C29H32FN7 O11Cu (%): C, 47.25; H, 4.38; N, 13.30; Cu, 8.62. Found (%): C, 47.20; H, 4.37; N, 13.29; Cu, 8.41. Λm (Ω^-1^mol^-1^ cm^2^) = 30; FT‐IR (ν, cm^-1^): (O-H/H2O) 3448, ν(C=O) pyridone 1626, ν(COO^-^)asym 1584, ν(COO^-^)sym 1384, (M-O) 520. UV–Vis (λmax, nm): 276, 342,418, 654. ESR: g1=1.97, g2=2.14, g3=2.57; ˂g˃= 2.23; Rr =0.40.

#### Fluorescence studies

Pefloxacin, a stock solution of 2.00x10^-7^ M, was synthesized by dissolving an accurate mass of pefloxacin in double-distilled water and recording its fluorescence intensity. Different concentrations of Cu(NO3)2.3H2O solutions (5.00x10^-3^ –1.00x10^-7^ M) were added to the solution, and their fluorescence intensity was measured.

#### Amino acid detection

Different concentrations of three amino acids, namely, aspartic acid, proline, and alanine (1.3x10^-1^ –1.00x10^-3^ M), were added gradually to the prepared copper complex solution. In this investigation, 3 mL of 0.01M phosphate buffer (pH 7) was added to the mixture.

The buffer solutions were synthesized by mixing the proper volumes of 0.1MNaOH and 0.01M KH2PO4 for pH 5–10. Immediately after sample preparation the spectra were recorded by scanning the wavelength range from 350 to 600 nm at an optimum excitation wavelength of 330 nm.

#### Computational methodology

The Gaussian 09 program [31] is a powerful software for the structural and electronic analysis of a large number of molecules. The molecular properties were studied using the B3LYP method [32, 33] with mixed basis set LanL2DZ for the metal and 6-311G(d,p) for C,H,O,N and F of the ligand [34, 35]. Full optimization was applied to pefloxacin and its binary and ternary copper(II) complexes to produce important geometrical parameters that indicate the actual structure of the synthesized complexes. A computational study was performed on gas state, quantum, and reactivity parameters, including frontier molecular orbitals (FMO) energies, electronegativity, ionization potential, electron affinity, global descriptors (chemical softness and hardness), and dipole moment. The Chemcraft [36] and Gauss view [37] programs envisage the calculated optimized structures and produce some calculations based on the FMOs. Natural bond orbital (NBO) analysis and molecular electrostatic potential (MEP) were studied for the optimized structures at the DFT/B3LYP level [32, 33]. To demonstrate the kind of interactions between the atoms of compounds, noncovalent interaction analysis was performed on the optimized compounds.

#### Antimicrobial activity studies

A modified Kirby-Bauer disc diffusion technique [38–41] was carried out to evaluate the antimicrobial activity of pefloxacin and its Cu(II) complexes against bacterial strains (*B. subtilis, S. pneumoniae, E. coli, and P. aeruginosa*) and against fungal strains (*C. albicans and A. fumigatus)*. A stock solution of 5 mg/mL prepared by dissolving pefloxacin and the copper complexes in dimethyl sulfoxide. Twofold dilution of the stock solution was carried out to obtain solutions of several concentrations. The antibacterial and antifungal activities of the investigated compounds were determined by the filter paper disc method [42], and the diameters of the inhibition zones (mm) were measured to evaluate the activities. Media with DMSO was used as a control. Standard discs of gentamicin and ampicillin (antibacterial agents; 10 μg/disc) and amphotericin B (antifungal agent; 10 μg/disc) served as positive controls for antimicrobial activity, while negative control was employed as a filter disc soaked with 10 µl of dimethyl sulfoxide.

#### Molecular docking simulation

Docking simulation was performed based on the crystal structure of *E. coli* and *S. pneumonia* receptors using 5I2D [43] and 6O15 [44] codes downloaded from the Protein Data Bank (PDB) (https://www.rcsb.org/structure).

The software iGemdock 2.1 [45] was used for the current calculations, and Chimera 1.13.1progrsm [46] was used for the visualization of ligand-target interactions. The docking performance has a default setting based on X-ray diffraction with 2.30 Å resolution and 0.193 R-factor. Docking accuracy settings (with GA parameters) were attuned as a standard docking with a size of population 200, generations 70 and number of solutions 2. The receptor preparation was performed by eliminating any ions, small ligands and water molecules and adding polar hydrogens.

## Results and discussion

The prepared copper(II) complexes (Scheme 1) are stable at room temperature, freely soluble in dimethyl sulfoxide but insoluble in most organic solvents and water. The detected lower molar conductivity value for [Cu(Pf)_2_(H_2_O)_2_].3H_2_O (3-4 S.cm^2^.mol^-1^) suggested a nonelectrolytic nature of the complex. The molar conductivity value of 20-50 S.cm^2^.mol^-1^ for [Cu(HPf)(bipy)(NO3)]NO3.2H2O and [Cu(HPf)(phen)(NO3)]NO3.2H2O indicated that both complexes are 1:1 electrolytes [47].

Diverse crystallization methods were carried out to gain a crystal appropriate for structure determination using X-ray crystallography. Nevertheless, the complexes were collected as microcrystalline products.

### FT-IR spectroscopy

The FT-IR spectra of the free ligand and its complexes showed a broad split band in the region 3450-3414 cm^-1^ corresponding to ν(O-H/H2O). The strong bands at 1718 and 1630 cm^−1^ in the spectrum of pefloxacin assigned to carboxylic and pyridone ν(C=O) moieties, respectively [27, 48]. However, in the spectra of complexes, the pyridone ν(C=O) was shifted, appearing in the range 1626-1635 cm^-1^, while carboxylic ν(C=O) disappeared, and asymmetrical and symmetrical vibrations bands for of ν(C=O) were displayed at 1568-1589 cm^-1^ and 1350-1386 cm^-1^.

The Δν values were 200-218, indicating monodentate binding to copper(II) ions. The ternary complexes [Cu(HPf)(bipy)(NO3)]NO3.2H2Oand [Cu(HPf)(phen)(NO3)]NO3.2H2O showed bands at 2712 and 2714 cm^-1^ respectively, corresponding to ν(H-N^+^) of the biperazine ring, indicating the neutral zwitterionic form of pefloxacin. Two new bands were shown at 1485,1386 and 1481,1384 cm^-1^ for [Cu(HPf)(bipy)(NO3)]NO3.2H2O and [Cu(HPf)(phen)(NO3)]NO3.2H2O due to binding of the nitrato group to the copper atom. The ΔνNO3 for these complexes are 99 and 97 cm^-1^, respectively, representing a monodentate coordination mode of the nitrate group to the copper(II) ion, whereas the strong sharp bands at 1386 and 1384 cm^-1^ for [Cu(HPf)(bipy)(NO3)]NO3.2H2O and [Cu(HPf)(phen)(NO3)]NO3.2H2O, respectively, revealed the presence of a free ionic nitrate group. The N‒N-chelating heterocycle rings generate bands characteristic of overlapping ν (C=N) and ν (C=C) stretching vibrations in the 1440–1600 cm^−1^ range. Furthermore, the out-of-plane vibration of the heterocyclic 1,10-phenanthroline ring in [Cu(HPf)(phen)(NO3)]NO3.2H2O appeared at 720 cm^-1^ while that to 2,2ʹ- bipyridine in [Cu(HPf)(bipy)(NO3)]NO3.2H2O was displayed at 779 cm^-1^ [19, 48], confirming the formation of mixed ligand complexes with distorted square-based pyramidal structures.

### UV‒Vis spectroscopy

The bands appeared at 283,340 and 415 nm in the spectrum of pefloxacin were assigned to π- π *, n- π * and charge transfer transitions, respectively [27]. Upon complex formation, these bands were variably shifted with increasing intensities. The spectrum of [Cu(Pf)2(H2O)2].3H2O showed one broad asymmetric absorption band at 723 nm ascribed to the [2]Eg → ^2^T_2_g transition, suggesting an octahedral configuration [49]. Furthermore, the bands at 649 and 654 nm for [Cu(HPf)(bipy)(NO3)]NO3.2H2O and [Cu(HPf)(phen)(NO3)]NO3.2H2O, respectively, are of penta-coordinated copper complexes having distorted square pyramidal geometry and are assigned to ^2^B_1_→ ^2^E_1_ transitions [50].

### Mass spectroscopy

The mass spectra of [Cu(Pf)_2_(H_2_O)_2_]0.3H_2_O, [Cu(HPf)(bipy)(NO_3_)]NO_3_.2H_2_O and [Cu(HPf)(phen)(NO_3_)]NO_3_.2H_2_O complexes **(**Additional file 1: Figures S1-S4**)** exhibited a peak with *m/z* 818, 713 and 737, respectively, consistent with their molecular weight, and their proposed fragmentation patterns are given in Schemes Additional file 1: S5- S8.

### EPR spectroscopy

The spectrum of [Cu(Pf)2(H2O)2].3H2O at room temperature showed axial parameters g||(2.31) ˃g┴ (2.07) ˃ge (2.0023) representing dx^2^-y^2^ ground state copper(II) complexes and gav of 2.13 [51]. The value of the G- parameter 4.52 indicated no copper-copper interaction in the solid-state and intermediate ligand field. The hyperfine line splitting factor A|| of 170 G obtained from the spectrum is consistent with the distortion from planarity. The empirical factor ƒ has a value of 136 cm^-1^ representing a minor distortion in the equatorial plane. The covalency parameter of 0.85 also indicates significant in-plane covalent σ bonding [52].

The spectrum of [Cu(HPf)(bipy)(NO3)]NO3.2H2O showed an axial shape with g||>g┴, gav> 2.0023, and G = 4.5 with no existence of hyperfine lines in the perpendicular or parallel regions. The EPR spectrum for [Cu(HPf)(phen)(NO3)]NO3.2H2O has a rhombic nature. The spectrum showed three g values g3(2.57)>g2(2.14)> g1(1.97). The average g value of 2.23 and R value of 0.40, confirmed the dx^2^-y^2^ ground state [51, 52].

### Fluorescence spectroscopy

**Fig. 1:**
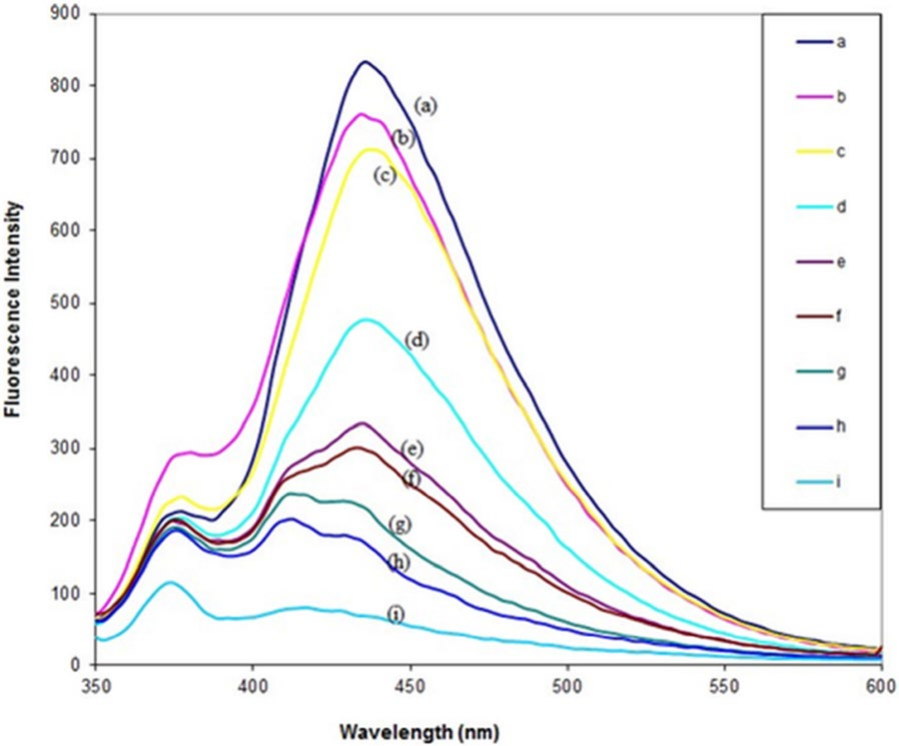
The effect of Cu^2+^ ions concentration (1.00 × 10^–7^–5.00 × 10^–3^ mol L^−1^) on the fluorescence intensity of pefloxacin (2.00 × 10^–7^ mol L^−1^) at 25 °C (λex at 330, λem at 435 nm) **a** [Hpf] = 2.00 × 10^–7^ mol L^−1^, **b** upon addition of [Cu^2+^] = 1.00 × 10^−7^ mol L^−1^, **c** upon addition of [Cu^2+^] = 4.00 × 10^–7^ mol L^−1^, **d** upon addition of [Cu^2+^] = 1.00 × 10^− 4^ mol L^−1^, **e** upon addition of [Cu^2+^] = 2.00 × 10^–4^ mol L^−1^, **f** upon addition of [Cu^2+^] = 3.00 × 10^–4^ mol L^−1^, **g** upon addition of [Cu^2+^] = 1.00 × 10^–3^ mol L^−1^, **h** upon addition of [Cu^2+^] = 2.00 × 10^–3^ mol L^−1^, **i** upon addition of [Cu^2+^] = 5.00 × 10^–3^ molL.^−1^

The fluorescence emission spectra of pefloxacin (2.00 × 10^–7^ mol L^−1^) in the absence and presence of various concentrations of Cu^2+^ ions (1.00 × 10^–8^-5.00 × 10^–3^ mol L^−1^) are given in Fig. 1. As shown from the displayed spectra, with an excitation wavelength of 330 nm, the maximum emission wavelength of pefloxacin was 435 nm (Fig. 1a). The fluorescence intensity of pefloxacin decreased with increasing Cu^2+^ ion concentration (Fig. 1b-i), but no change in the maximum emission wavelength of HPf was observed. This observation could be attributed to the high affinity of Cu^2+^ ions to the carbonyl and carboxylic groups present in the pefloxacin molecule as a ligand [53] and that the ion can strongly quench the inner fluorescence of the pefloxacin ligand and that the interaction between the bioligand and metal ion indeed existed without inducing any conformational change in pefloxacin. Static quenching implies either the existence of a sphere of effective quenching or the formation of a ground state nonfluorescent complex. The observed static quenching mechanism of fluorescence was confirmed by applying Stern–Volmer technique [54] and calculating the quenching constant (Ksv) utilizing Eq. (1) at 25 °C and 35 °C. If Ksv decreases with increasing temperature, it is concluded that the quenching process is static rather than dynamic [55].

1 $${\text{F}}_{0} /{\text{F}} = { 1 } + {\text{ Ksv}}[{\text{Cu2}} + ]$$

Herein, F0 and F are the fluorescence intensities of the bioligand in the absence and presence of the Cu^+2^ quencher, respectively. The value of Ksv was calculated from the slope of the linear plot of F0/F vs [Cu^2+^] (Additional file 1: Figure S9).

From the experimental data in Additional file 1: Figure S9, it is clear that increasing temperature led to the decreased in K_sv_ value (K_sv_ = 0.151 × 10^4^ Lmol^−1^ at 25 °C and 0.146 × 10^4^ Lmol^−1^ at 35 °C). Inconclusion, quenching is typically initiated by static processes. Copper ions are known as strong quenchers because of their electronic structure (d^9^). Quenching by this type of substance most likely involves the donation of an electron from the fluorophore to the quencher.

Cu^2+^ usually presents low energy levels, which give rise to energy and electron transfer processes and can quench the fluorescent excited state of the molecule [55].

### Detection of amino acids

**Fig. 2:**
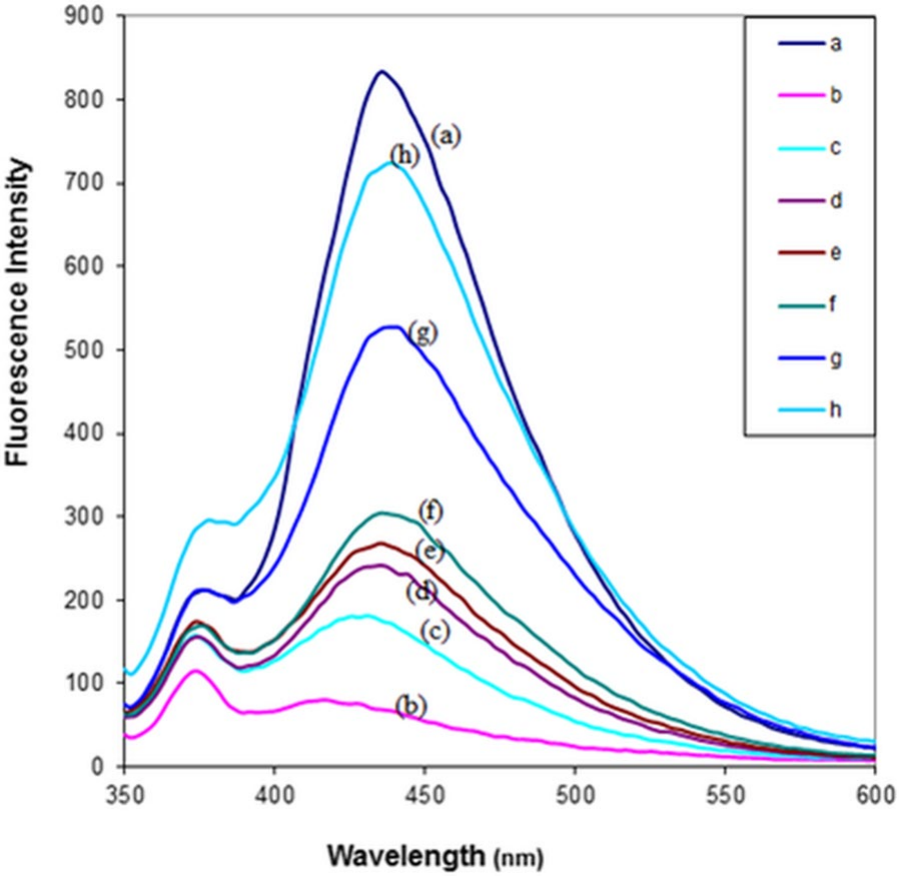
Changes of fluorescence intensity after addition of different concentrations of aspartic acid (asp) in 0.01 mol L^−1^ phosphate buffer solution (pH 7). [HPf] = 2.00 × 10^–7^ mol L^−1^, **b** upon the addition of [Cu^2+^] = 5.00 × 10^–3^ mol L^−1^, **c** upon the addition of [Cu^2+^] = 5.00 × 10^–3^ mol L^−1^ and [asp] = 2.00 × 10^–3^ mol L^−1^, **d** upon the addition of [Cu^2+^] = 5.00 × 10^–3^ mol L^−1^ and [asp] = 4.00 × 10^–3^ mol L^−1^, **e** upon the addition of [Cu^2+^] = 5.00 × 10^–3^ mol L^−1^ and [asp] = 6.00 × 10^–3^ mol L^−1^, **f** upon the addition of [Cu^2+^] = 5.00 × 10^–3^ mol L^−1^ and [asp] = 1.00 × 10^–2^ mol L^−1^, **g** upon the addition of [Cu^2+^] = 5.00 × 10^–3^ mol L^−1^ and [asp] = 3.00 × 10^–2^ mol L^−1^, **h** upon the addition of [Cu^2+^] = 5.00 × 10^–3^ mol L^−1^ and [asp] = 4.50 × 10^–2^ mol L^−1^ The acidic conditions were avoided during detection, and phosphate buffer solution at pH = 7 was used to allow the stability of the complex. Thus, amino acids act as competitors, as they reduce the quenching effect of Cu^2+^ ions from reacting with pefloxacin due to the development of a Cu–amino acid complex

To assess the copper-pefloxacin complex as a fluorescent probe for detecting various amino acids, several concentrations of amino acids, namely, alanine, aspartic acid, and proline, in 0.01 mol L^−1^ phosphate buffer solution (pH 7) were added to the nonfluorescent copper-pefloxacin complex solution. Figure 2 represents the changes in fluorescence intensity after the addition of different concentrations of aspartic acid (asp) in 0.01 mol L^−1^ phosphate buffer solution (pH 7), while the changes in the intensity of fluorescence spectra after the addition of different concentrations of proline and alanine are provided in the supplementary information **(**Additional file 1: Figures S10 and S11). When the amino acid was added to a solution of copper-pefloxacin complex, the pefloxacin in the copper-pefloxacin complex was replaced with the added amino acid forming a copper-amino acid complex. Consequently, quenching by copper(II) ions was inhibited, and the emission from pefloxacin was amplified drastically [56]. The suggested mechanism for amino acid determination was explained in equations.

2 $${\text{Cu}}^{{2^{ + } }} + {\text{ HL }} \to \, \left[ {{\text{Cu}}{-}{\text{L}}} \right] + {\text{ H}}$$

3 $$\left[ {{\text{Cu}}{-}{\text{L}}} \right] \, + {\text{ amino acid }} \to \, \left[ {{\text{Cu}}{-}{\text{amino acid}}} \right] + {\text{L}}$$

The relative fluorescence intensity change for copper-pefloxacin complexes at 435 nm after the addition of 4.50 × 10^–2^ mol L^−1^ of different amino acids to 0.01 mol L^−1^ phosphate buffer solution containing [Cu^2+^] = 5.00 × 10^−3^ mol L^−1^and [Hpf] = 2.00 × 10^–7^ mol L^−1^for a pefloxacin solution as a probe is displayed in Additional file 1**:** Figure S12. Based on the obtained results, it is obvious that the rise in the intensity of fluorescence upon adding aspartic acid is notable from other amino acids. This may be due to the overall stability sequence of the copper complexes with amino acids, which follows the order aspartic acid < proline < alanine. Thus, aspartic acid can be simply determined fluorometrically upon using copper-pefloxacin solution probe. A good linear relationship between the concentration of aspartic acid, proline, and alanine and the fluorescence intensity was observed from the calibration curves of amino acids in pefloxacin solution at λmax 435 nm in 0.01 mol L^−1^ phosphate buffer solution (pH 7) at 298 K **(**Additional file 1: Figure S13). The linear range of the calibration curves for the pefloxacin solution was 2.00 × 10^–3^–4.5 × 10^–2^ mol L^−1^, 1.00 × 10^–2^– 4.5 × 10^–2^ mol L^−1^, and 2.00 × 10^–2^ –1.3 × 10^–1^ mol L^−1^ for aspartic acid, proline, and alanine, respectively. In conclusion, pefloxacin has a high fluorescence intensity, while copper- pefloxacin complex is weaker than copper-amino acid complexes. Thus, pefloxacin can be used as a probe for amino acids detection by copper ions.

### Thermal analysis

Thermogravimetric analysis (TGA), derivative thermogravimetric analysis (DTG), and differential thermal analysis (DTA) investigated the thermal analysis of pefloxacin and its copper(II) complexes in a stream of nitrogen. Additionally, the % weight losses during the degradation stages were determined and predicted in terms of the molecular formula and are given in Table 1. The thermogravimetric analysis curve of pefloxacin mesylate dihydrate showed a strong degradation stage in the temperature range of 46-999 °C with a mass loss of 37.907% (calc. 37.834%) due to the loss of 2H2O, C2H4O13 and CO2. The residual solid corresponding to a mass of 62.093% (calc. 62.149%), which is equivalent to the C16H20FN3O residue. This step is accompanied by three exothermic DTA peaks at 60 °C, 143 °C and 234 °C.

**Table 1:** Thermal analysis of pefloxacin and its complexes

Compound	Steps	T (°C)	% wt loss Found(Calc.)	Fragment	T (°C)
Pefloxacin mesylate dihydrate	I	46–999	37.907(37.834)	C_2_H_4_O_5_S + 2H_2_O	536
			62.093(62.149)	Residue (C_16_H_20_FN_3O_)	
[Cu(Pf)_2_(H_2_O)_2_].3H_2_O	I	47–116	6.188(6.599)	3 H_2_O	75
	II	182–285	28.342(28.366)	2 (H_2_O + C5H_10_N_2_)	248
	IIII	285–586	57.450 (57.004)	2(C_12_H_8_FNO_3_)	513
			7.797(7.765)	Residue (Cu)	
[Cu(HPf)(bipy)(NO_3_)]NO_3_.2H_2_O	I	28–282	50.661(50.507)	2H_2_O + 2NO_3_ + CO_2_ + C_10_H_8_N_2_ CO_2_ + C_11_H_8_N_2_O_2_	272
	II	282–482	36.191(36.598)	C_15_H_20_FN_3_	439
			13.148(12.837)	Residue (CuO + C)	
[Cu(HPf)(phen)(NO_3_)]NO_3_.2H_2_O	I	56–264	27.305(27.675)	2H_2_O + 2NO_3_ + CO_2_	257
	II	264–378	16.948(17.093)	C_7_H_14_N_2_	344
	III	378–647	45.354(44.362)3)	C_21_H_14_FN_3_	509
			10.393(10.785)	Residue (CuO)	

The three copper complexes displayed nearly similar types of thermal decomposition [57, 58]. The degradation continued, leaving copper metal in the binary complex and copper oxide in both ternary complexes as the end product. The observed results were in accordance to the proposed structures of the complexes.

### Determination of thermodynamic parameters for Pefloxacin and its Cu(II) complexes

The thermodynamic parameters of the degradation process of pefloxacin and its copper(II) complexes, including the activation energy (Ea), enthalpy change (ΔH), and entropy change (ΔS), were estimated by performing the Horowitz-Metzger Eq. ^59^ and are listed in Additional file 1: Table S14. The order of the chemical reaction (n) was calculated by the peak symmetry method [60]. The values of the collision factor (Z) [61] were calculated from the relation:

4 $$Z = \frac{{E_{a} }}{{(RT_{m} )}}\phi \,exp\,\left( {\frac{{E_{a} }}{{(RT_{m}^{2} )}}} \right) = \,\frac{{KT_{m} }}{h}exp\,\left( {\frac{\Delta S}{R}} \right)$$

where (ΔS), (R), (ϕ) (K) and (h) represent the entropies of activation, molar gas constant, rate of heating (K S^-1^), Boltzmann constant, and Planck's constant, respectively [61]. The change in enthalpy (ΔH) for any phase transformation taking place at any peak temperature (Tm) can be given by the following equation (ΔS=ΔH/T) [62]. Based on the least square calculations, the LnΔT versus 1000/T plots for all complexes gave straight lines from which the activation energies were calculated according to the method of Piloyan et al. [63]**.** The slope is of the Arrhenius type and equals –**Ea**/R. With the foregoing discussion in mind, the TGA/DTG and TGA/DTA measurements revealed that copper complexes of pefloxacin undergo decomposition to form copper oxide as the final product except for [Cu(Pf)_2_(H_2_O)_2_].3H_2_O complex that undergoes decomposition to form elemental copper as a final product. The data resulting from the DTA curves revealed that the maximum and minimum values of the collision number (Z) are 2579 and 76, respectively, suggesting different mechanisms with variable speeds. Additionally, the values of the decomposed substance fraction (αm) at the maximum development of the reaction in each step are of similar magnitude and lie within the range 0.637–0.423. In addition, the (∆S) values for pefloxacin and its complexes are in the range of -0.184 to -0.218 kJK^-1^mol^-1^. The negative sign indicates that the transition states are more ordered and in a less random molecular configuration than the reacting complexes [64–66].

Finally, the (n) values suggest that the reaction is incomplete and/or proceeds in a complicated mechanism [67] the negative values of (∆H) indicate exothermic decomposition processes.

### DFT calculations

#### Optimization and geometrical structure

**Fig. 3:**
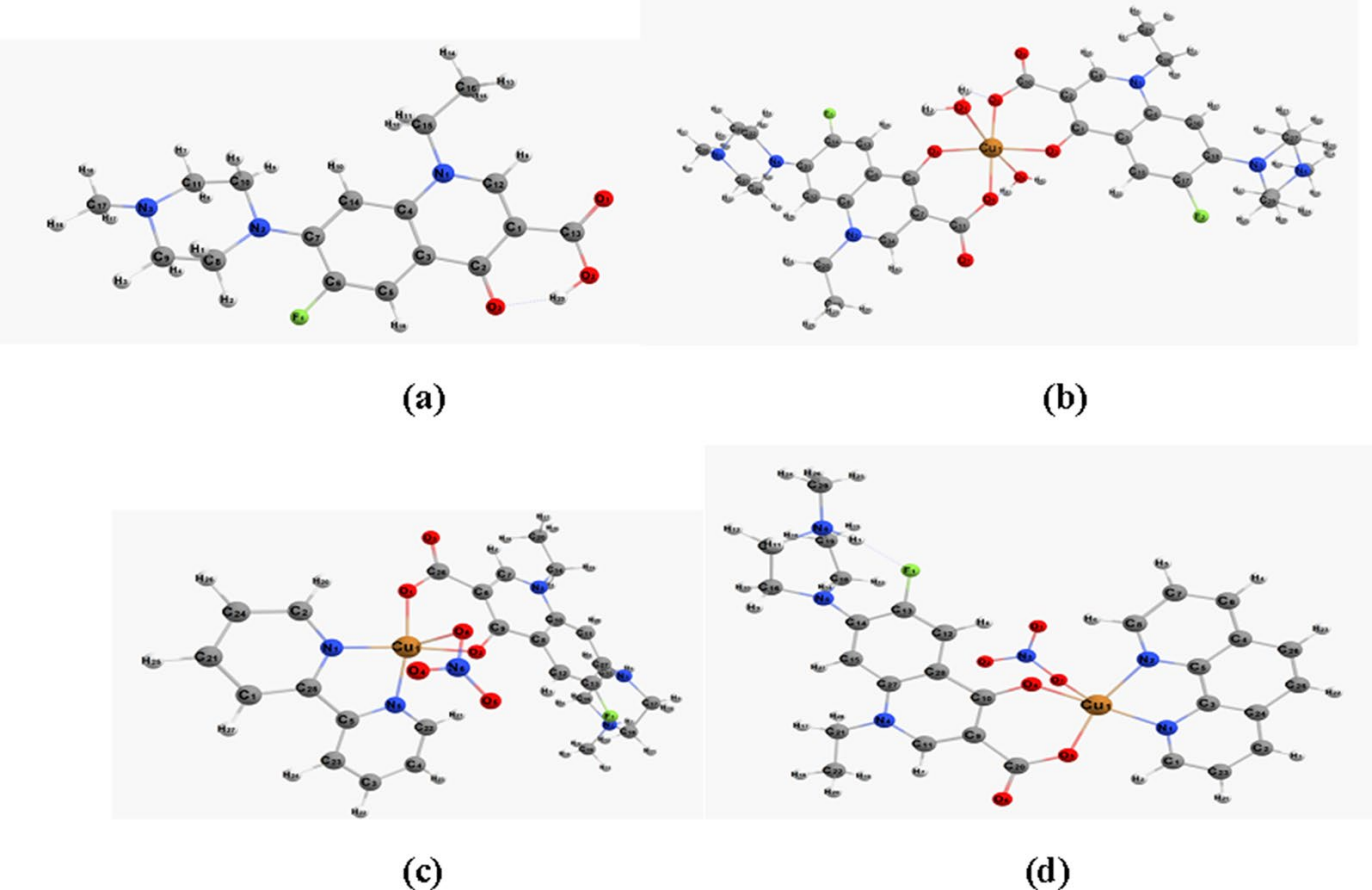
The optimized structures of (**a**) pefloxacin (**b**) [Cu(Pf)_2_(H_2_O)_2_].3H_2_O (**c**) [Cu(HPf)(bipy)(NO_3_)]NO_3_.2H_2_O (**d**) [Cu(HPf)(phen)(NO_3_)]NO_3_.2H_2_O, using DFT/B3LYP/LanL2DZ/6-311G(d,p) method

The optimized geometrical structures of pefloxacin and its complexes are displayed with numbering systems in Figure 3 The geometrical parameters (bond lengths and bond angles) are computed and tabulated in Table 2. In the [Cu(Pf)2(H2O)2].3H2O complex, the copper atom is coordinated with two water molecules and two bidentate pefloxacin molecules, forming a six-coordinate octahedral complex. The bond lengths around the Cu atom refer to the group axial position, where Cu1-O1 andCu1-O8 are 2.369 Å and 2.425 Å, respectively. The two H-bonding bonds C2---H1 and C5--- H41 with bond lengths of 2.627 Å and 2.712 Å, respectively, can enhance the stability of complex formation.

**Table 2:** Theoretical geometric parameters (bond lengths and bond angles) for copper (II) based pefloxacin complexes

HPf		[Cu(Pf)_2_(H_2_O)_2_].3H_2_O		[Cu(HPf)(bipy)(NO_3_)]NO_3_.2H_2_O		[Cu(HPf)(phen)(NO_3_)]NO_3_.2H_2_O	
(a) Bond Length (Å)							
C15-O20	1.246	Cu1-O1	2.369	Cu1-O2	2.145	Cu1-O3	2.249
C15-O21	1.365	Cu1-O2	1.958	Cu1-O4	1.908	Cu1-O4	1.993
O21-H44	1.016	Cu1-O3	1.955	Cu1-O5	1.927	Cu1-O5	1.944
C2-O22	1.660	Cu1-O4	2.004	Cu1-N1	2.002	Cu1-N1	2.0185
C18-N5	1.495	Cu1-O5	1.946	Cu1-N6	2.010	Cu1-N2	2.066
C7-F24	1.407	Cu1-O8	2.425	C26-O4	1.337	C14-N5	1.428
C8-N13	1.397	C17-F2	1.409	C9-O5	1.310	N4-C21	1.500
C23-N17	1.473	C14-F1	1.406	C13-F1	1.404	O6-C20	1.254
O22–-H44	1.660	O5–-H41	2.627	N3-C14	1.501	C13-F1	1.430
		O2–-H1	2.712				
(b) Bond Angle (^o^)							
O1-C13-O2	122.39	O1-Cu1-O2	73.46	O2-Cu1-O4	92.53	O3-Cu1-O4	87.84
C13-O2-H20	110.87	O1-Cu1-O3	109.49	O2-Cu1-O5	102.56	O3-Cu1-O5	110.34
C12-N1-C15	121.50	O1-Cu1-O4	86.91	O2-Cu1-N1	95.88	O3-Cu1-N1	97.22
C11-N3-C17	112.98	O1-Cu1-O5	109.02	O2-Cu1-N6	87.43	O3-Cu1-N2	90.04
C5-C6-F1	117.72	O1-Cu1-O8	174.30	N1-Cu1-N6	81.54	O4-Cu1-O5	91.68
C7-N2-C10	119.66	O2-Cu1-O3	91.06	N1-Cu1-O4	93.57	O4-Cu1-N1	174.12
		O2-Cu1-O4	91.32	N1-Cu1-O5	167.52	O4-Cu1-N2	95.69
		O2-Cu1-O5	177.12	N6-Cu1-O4	167.51	O5-Cu1-N1	89.34
		O2-Cu1-O8	102.45	N6-Cu1-O5	93.65	O5-Cu1-N2	158.58
		O3-Cu1-O4	163.41	O4-Cu1-O5	93.34	N1-Cu1-N2	81.39
		O3-Cu1-O5	86.73				
		O3-Cu1-O8	66.21				
		O4-Cu1-O5	90.27				
		O4-Cu1-O8	97.25				
		O5-Cu1-O8	74.96				

The bond angles around the copper center involved the presence of the complex in the distorted octahedral structure. However, in [Cu(HPf)(bipy)(NO3)]NO3.2H2O, the copper atom is coordinated to O4 and O5 from pefloxacin with bond lengths of 1.908 Å and 1.927 Å, respectively, and to O2 of the nitrate group, in addition to N1 and N6 from the bipyridine ligand at 2.002 Å and 2.010 Å, respectively, to complete the five-coordinate copper(II) complex. The bond length values confirmed the axial position of O2 due to bond elongation. The bond angles of a copper basal plane (between Cu and O4, O5, N1, and N6 atoms) indicate distorted square pyramidal geometry. For the [Cu(HPf)(phen)(NO3)]NO3.2H2O complex, the copper atom is coordinated to O3 of the nitrate molecule, O4, and O5 of the pefloxacin molecule in addition to N1 and N2 of the phenanthroline molecule. The bond length of Cu1-O3 is 2.249 Å, indicating the axial position of the nitrate group, and the bond angles around the copper confirmed its distorted square pyramidal geometry.

### Quantum and reactivity parameters

To determine the electronic behavior of pefloxacin and its copper(II) complexes, the chemical reactivity parameters, including Frontier molecular orbital (FMO) energies, electronegativity (χ), ionization potential (I), electron affinity (A), dipole moment (D), chemical potential (µ), chemical hardness (η) and global softness (S), are calculated and given in Table 3.

**Table 3:** Quantum and reactivity parameters of pefloxacin and its copper(II) complexes

Parameter	HPf	[Cu(Pf)2(H2O)2].3H2O	[Cu(HPf)(bipy)(NO3)]NO3.2H2O	[Cu(HPf)(phen)(NO3)]NO3.2H2O
E (a.u.)	− 1149.483	− 2646.856	− 2121.100	− 2197.291758
D (debye)	13.613	6.990	10.019	9.941
EHOMO (ev)	− 8.136	− 7.980	− 7.489	− 7.435
ELUMO (ev)	− 2.279	− 3.870	− 3.945	− 3.646
ΔE (ev)	5.857	4.110	3.544	3.789
I (ev)	8.136	7.980	7.489	7.435
A (ev)	2.27	3.870	3.945	3.646
η (ev)	2.929	2.055	1.772	1.894
µ (ev)	− 5.203	− 5.925	− 5.717	− 5.540
S (ev)	0.171	0.243	0.282	0.264
χ (ev)	5.203	5.925	5.717	5.540
ω (ev)	4.461	8.542	9.222	8.100

FMOs mainly represent two types of orbitals, the highest occupied molecular orbital (HOMO) and the lowest unoccupied molecular orbital (LUMO), that verify the reactivity and stability of compounds.

The high value of EHOMO represents the ease of the compound donating an electron to the unoccupied orbital of the molecule that acts as a receptor, while the low ELUMO value explains the small resistance of the molecule to accepting electrons. The energy gap (ΔE) is the difference between FMO (HOMO-LUMO) energies.

Both the I and the A values are related to EHOMO and ELUMO, which are helpful parameters in predicting the ability of the molecule to donate or accept electrons. As the studied complexes have lower I and higher A (3.945 eV) than the corresponding pefloxacin ligand, the complexes may act as electron donor systems.

The binary [Cu(Pf)2(H2O)2].3H2O complex showed a higher ΔE (4.110 eV), indicating higher stability and forming a complex with lower reactivity. In contrast, the [Cu(HPf)(bipy)(NO3)]NO3.2H2O complex has a lower energy gap (3.544 eV), demonstrating higher reactivity and lower stability. The global reactivity descriptors (chemical hardness and global softness) are based on the I and A parameters. A higher η (2.055 eV) and lower S (0.243 eV) for the [Cu(Pf)2(H2O)2] complex.3H2O indicates its lower reactivity [68]. The electrophilicity index ω measures the energy change when the system becomes saturated with electrons and is given by ω = μ^2^/2η. The global softness is calculated from S = 1/2η**.**

**Fig. 4:**
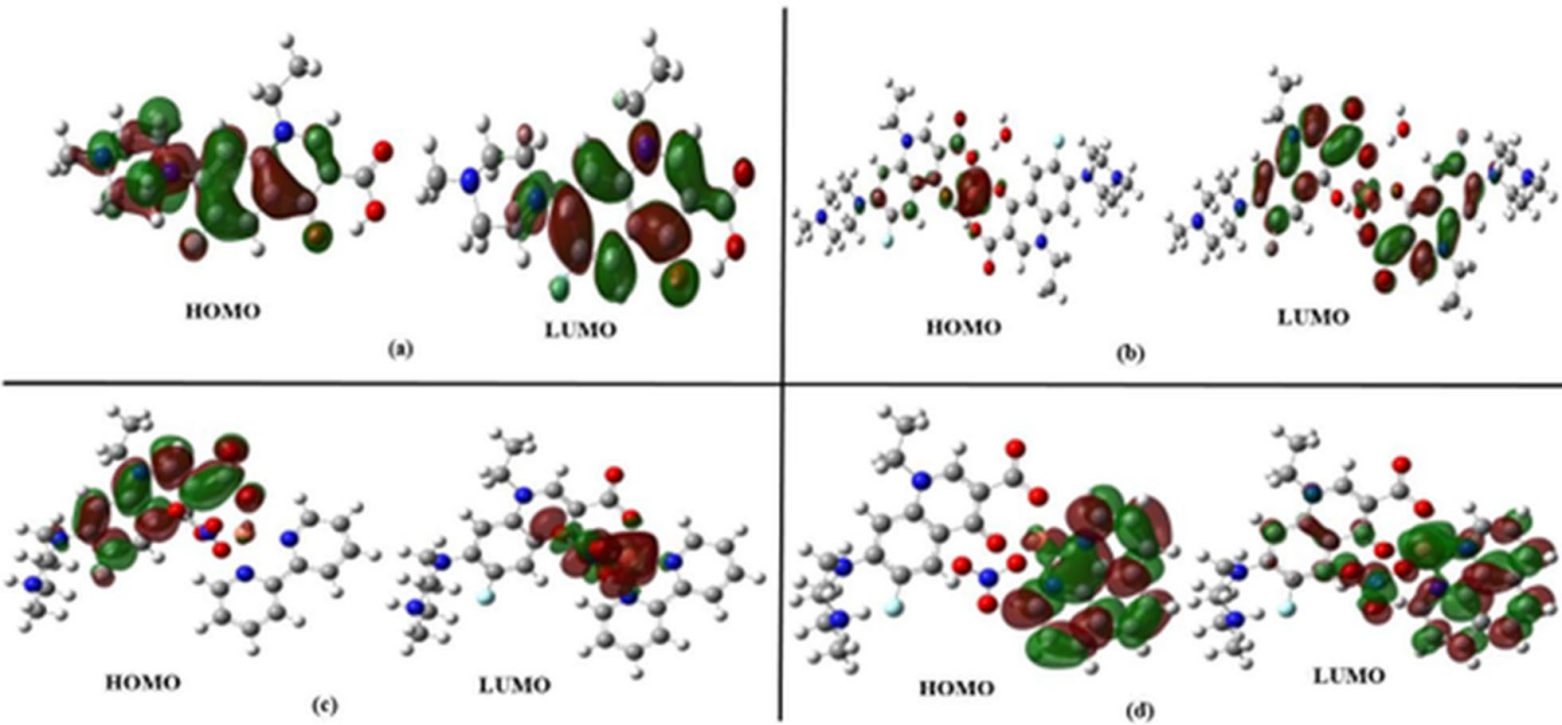
Electronic distribution of FMOs for the optimized compounds where **a** pefloxacin **b** [Cu(Pf)2(H2O)2].3H2O **c** [Cu(HPf)(bipy)(NO3)]NO3.2H2O **d** [Cu(HPf)(phen)(NO3)]NO3.2H2O

Figure 4 shows that the electronic orbital densities dispersed on the HOMO and LUMO of the examined complexes involve the copper center coordinated with the ligand.

This dispersion ensures the possible transition of electrons from the HOMO to LUMO levels. The following equations describe the calculated parameters: [69]

5 $${\text{Ionization potential }}\left( {\text{I}} \right) = \, - {\text{EHOMO}}$$

6 $${\text{Electron affinity }}\left( {\text{A}} \right) = - {\text{ELUMO}}$$

7 $${\text{Chemical hardness }}\left( \eta \right) \, = \left( {{\text{I}} - {\text{A}}} \right)/{2}$$

8 $${\text{Chemical potential }}\left( \mu \right) \, = \, - \left( {{\text{I }} + {\text{A}}} \right)/{2}$$

9 $${\text{Electronegativity }}\left( \chi \right) \, = \, - ({\text{EHOMO}} + {\text{ELUMO}})/{2}$$

10 $${\text{Electrophilicity index}}\left( \omega \right) = \mu {2}/{2}\eta$$

11 $${\text{Global softness }}\left( {\text{S}} \right) \, = {1}/{2}\eta$$

### Natural bond orbital (NBO) analysis and molecular electrostatic potential (MEP) profiles

The natural charge on each atom (except Hydrogens) of pefloxacin and its complexes after optimization are compiled in Additional file 1**:** Table S15, where the charge value predicts the active sites in the molecule. The charges on O2 and O3 of pefloxacin are − 0.364 and—0.341, respectively. This relatively high electronic charge indicates that the coordinative ligand atoms with the metal form a stable complex. The reactivity of the studied complexes can be described by the analysis of MEP to perceive the intra- and intermolecular interactions in the investigated compounds. Different color codes appear in this MEP analysis evaluating the active sites in the compound.

**Fig. 5:**
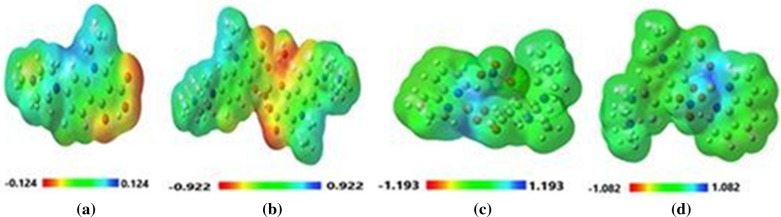
MEP surface of the optimized structures (**a**) pefloxacin (**b**) [Cu(Pf)2(H2O)2].3H2O (c) [Cu(HPf)(bipy)(NO3)]NO3.2H2O (d) [Cu(HPf)(phen)(NO3)]NO3.2H2O

The colored map of MEP for pefloxacin and its complexes are described in Fig. 5**,** where the electron density ranged from red to blue, corresponding to the highest and lowest electron density on the surface. In pefloxacin, the negative potential is attributed to O1, O2, and O3 atoms, which represent the active sites for coordination with the metal ion to form the complex, confirming the same results as in the NBO population. MEP of [Cu(Pf)2(H2O)2]0.3H2O indicated that the charge density delocalized on oxygen atoms of water and oxygen atoms of pefloxacin surround the copper center, which supports the electron-rich coordination region and stabilizes the electronic system.

In the case of complexes Cu(HPf)(bipy)(NO3)]NO3.2H2O and [Cu(HPf)(phen)(NO3)]NO3.2H2O, the electron density appeared to be poor around the coordination sphere, which may be attributed to the zwitter ion present in the complex that makes the surface potential on the surface mainly positive.

### Topological properties of Pefloxacin and [Cu(Pf)2(H2O)2]0.3H2O (NCI-RDG analysis)

Noncovalent bond interactions (NCIs) between diverse parts of the compound were detected using reduced density gradient (RDG) analysis. To apply RDG analysis in our study, the pefloxacin ligand and [Cu(Pf)_2_(H_2_O)_2_].3H_2_O complex were selected to perform this topological analysis. The different noncovalent interactions presented different color codes [70, 71] where the blue color represents a strong HB attraction that occurs between the oxygen of quinolone C=O and the carboxylic OH. In addition to the coordinated ligand sites that occur in the complex, there is a noncovalent HB interaction around the metal ion involving the bonding of water hydrogens with the coordinated pefloxacin oxygen atoms that describes the extensive stability of the complex. The green color represents electrostatic (vdW) interactions, while the red color indicates strong repulsion between the entries of the molecules distributed on different regions of pefloxacin and its copper complex [Cu(Pf)_2_(H_2_O)_2_].3H_2_O.

**Fig. 6:**
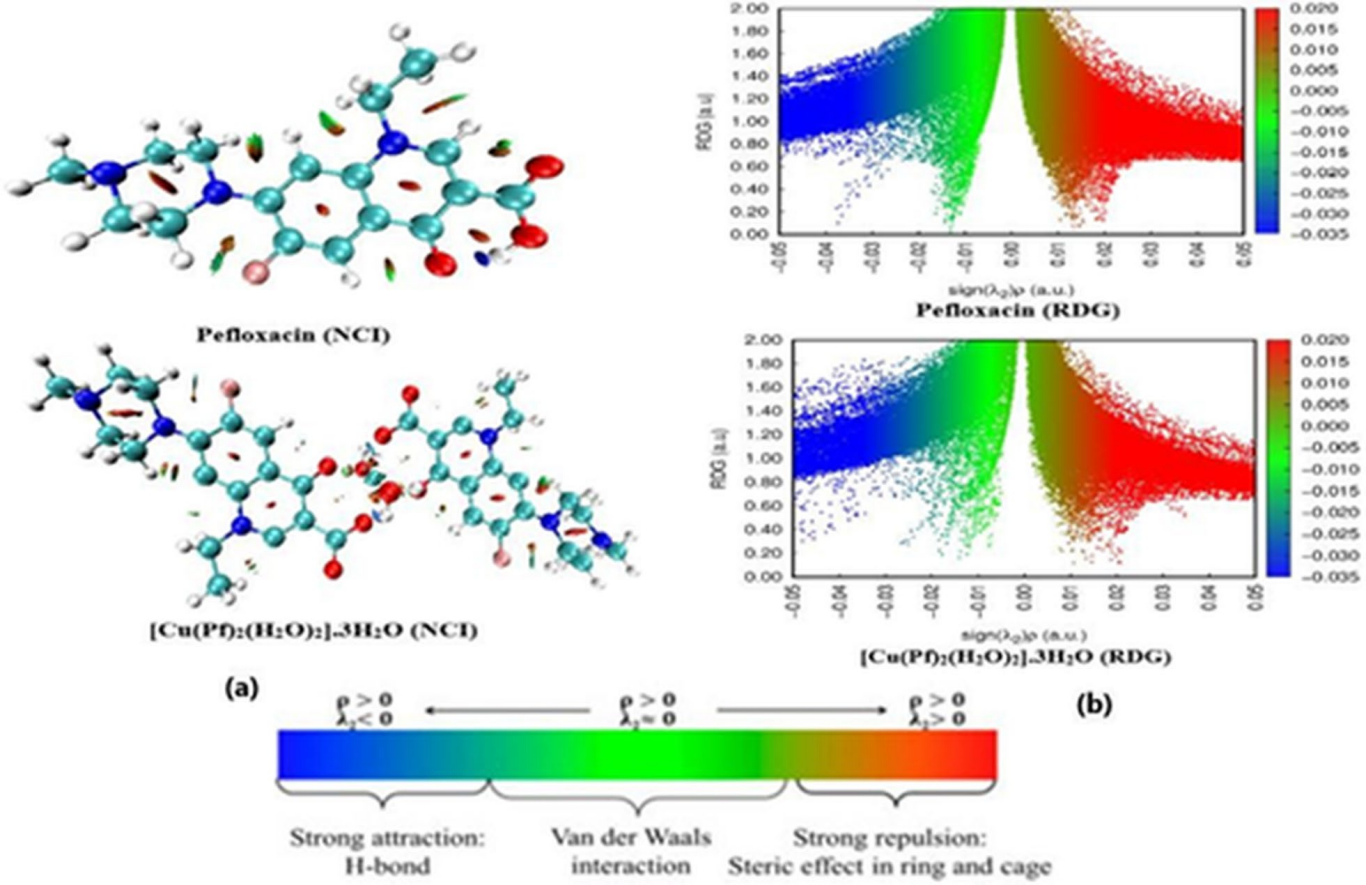
NCI isosurfaces **a** and RDG scatter mapping diagrams **b** of the optimized pefloxacin and its binary complex

The sign (λ2)ρ is a parameter describing the multiplication of the electron density with the sign of the second Hessian eigenvalue; it measures the strength of HB interaction in compounds and distinguishes this type from other interactions. The most negative sign (λ2)ρ value expresses strong HB (blue color), and the most positive sign (λ2)ρ value is evidence of strong steric interaction. As shown in Figure 6 plot (b) appeared as three spikes. In the case of pefloxacin, the low negative density gradient spike (sign(λ2)ρ < -0.03) corresponded to intramolecular pefloxacin H-bonding, while the second negative spike (0< sign(λ2)ρ > -0.03) corresponded to the van der Waals interactions that mostly occurred in the nonpolar hydrocarbons. When the spike moved to a positive RDG area (sign(λ2)ρ < 0.01), a strong repulsion interaction mostly occurred between the aromatic rings of the compound. In the case of [Cu(Pf)_2_(H_2_O)_2_].3H_2_O, the H-bonding density gradient spike is shifted to a lower negative value (sign(λ2)ρ< -0.04) due to the formation of several HBs during metal-ligand complexation. Additionally, the second negative spike of the van der Waals interaction appears to be dispersed with a lower density gradient value.

### Antimicrobial activities

Pefloxacin and its three complexes were executed in vitro against six pathogenic microorganisms*, two* gram-positive bacteria: *B. subtilis* and *S. pneumoniae*, two gram-negative bacteria *E. coli* and *P. aeruginosa,* two pathogenic fungi: *C. albicans* and *A. fumigatus* whereas Gentamicin and Ampicillin worked as standard bactericides and amphotericin B served as a fungicide.

The susceptibility of the pathogenic microorganisms to the compounds was refereed by measuring the diameter of the inhibition zone, and the data are summarized in Table 4. The bacterial screening data revealed that pefloxacin and its Cu(II) complexes exhibited activity against *Escherichia coli* and displayed better activity than Gentamicin. However, no activity was demonstrated against *P.aeruginosa*. The antibacterial activities were well-ordered as follows:

$$[{\text{Cu}}({\text{Pf}})_{2} ({\text{H}}_{2} {\text{O}})_{2} ] \cdot {3\text{H}}_{2} {\text{O}} > {\text{HPf}} > [{\text{Cu}}({\text{HPf}})({\text{bipy}})({\text{NO}}_{3})]{\text{NO}}_{3} \cdot {2\text{H}}_{2} {\text{O}} > [{\text{Cu}}({\text{HPf}})({\text{phen}})({\text{NO}}_{3})]{\text{NO}}_{3} \cdot {2\text{H}}_{2} {\text{O}}$$

**Table 4:** Antimicrobial screening results of pefloxacin and its complexes evaluated by the mean inhibition zone in mm

Compound	Gram negative bacteria		Gram positive bacteria		Fungi	
	*Escherichia coli*	*Pseudomonas aeruginosa*	*Streptococcus pneumonia*	*Bacillus subtilis*	*Aspergillus fumigatus*	*Candida albicans*
Pefloxacin mesylate dihydrate	22.4 ± 0.63	nda	21.2 ± 0.63	23.2 ± 0.63	nda	nda
[Cu(Pf)_2_(H_2_O)_2_].3H_2_O	22.1 ± 0.72	nda	24.3 ± 1.2	25.4 ± 0.58	18.3 ± 0.63	nda
[Cu(HPf)(bipy)(NO_3_)]NO_3_.2H_2_O	21.6 ± 0.63	nda	19.6 ± 1.2	20.8 ± 0.63	nda	nda
[Cu(HPf)(phen)(NO_3_)]NO_3_.2H_2_O	20.3 ± 0.63	nda	18.3 ± 0.58	18.9 ± 0.25	nda	nda
Amphotericin B					23.7 ± 0.1	25.4 ± 0.1
Ampicillin			23.8 ± 0.2	32.4 ± 0.3		
Gentamicin	19.9 ± 0.3	17.3 ± 0.1				

nda = no detected activity

The binary pefloxacin-Cu complex exhibited improved antibacterial activity compared to pefloxacin and the corresponding ternary complexes. Enhanced activity was observed against *S. pneumonia* compared to ampicillin. It was observed that pefloxacin and its ternary complexes did not show any noticeable antifungal activity, while the binary complex [Cu(Pf)_2_(H_2_O)_2_]0.3H_2_O showed moderate activities against *A. fumigates*. Based on the screened results, it is obvious that the [Cu(Pf)_2_(H_2_O)_2_]0.3H_2_O complex showed higher activity than free pefloxacin against some of the screened pathogenic microorganisms. This may be elucidated by chelation theory and overtone's concept [72–75].

The complex enhanced the lipophilic character of the central copper atom, that favors its permeation via the lipid bilayer of the microorganism's membrane and blocked the metal-binding sites on the enzymes of the microorganism, thus destroying them more aggressively. Reportedly, metal complexes have exclusive modes of action: ligand exchange or release, catalytic generation of toxic species, redox activation and/or depletion of essential substrates. Such mechanisms are hard or otherwise impossible to replicate with organic ligands [76].

Nevertheless, the effectiveness of complexes in several organisms depends on the impermeability of the cells of the microbes or on the changes in ribosomes in microbial cells [77]. The binary octahedral complex [Cu(Pf)_2_(H_2_O)_2_]0.3H_2_O demonstrated great effectiveness relative to the other prepared five-coordinate distorted square-based pyramidal complexes, [Cu(HPf)(bipy)(NO_3_)]NO_3_.2H_2_O and [Cu(HPf)(phen)(NO_3_)]NO_3_.2H_2_O. This may be due to its capability of forming strong M-L bonds which enhance its lipophilic character and its penetration into the lipid membrane, causing restriction in the growth of the microorganism. This outcome may be ascribed to its high coordination number and/or its octahedral geometry**.**

The change in the antimicrobial activity between the two prepared five-coordinate distorted square-based pyramidal copper complexes may be due to the change of heterocyclic rings involved in the coordination sphere [78]. However, [Cu(HPf)(bipy)(NO_3_)]NO_3_.2H_2_O demonstrated improved activity against the studied microorganisms compared with [Cu(HPf)(phen)(NO_3_)]NO_3_.2H_2_O. Surprisingly, these observations are not consistent with those in reported studies [19, 24, 79], which stated that mixed ligand complexes with 1,10-phenanthroline as a secondary ligand manifested improved antibacterial activity compared with other nitrogen donor heterocyclic ligands, owing to the nuclease activity of this ligand when complexed to copper.

### Protein binding screen evaluation

**Fig. 7:**
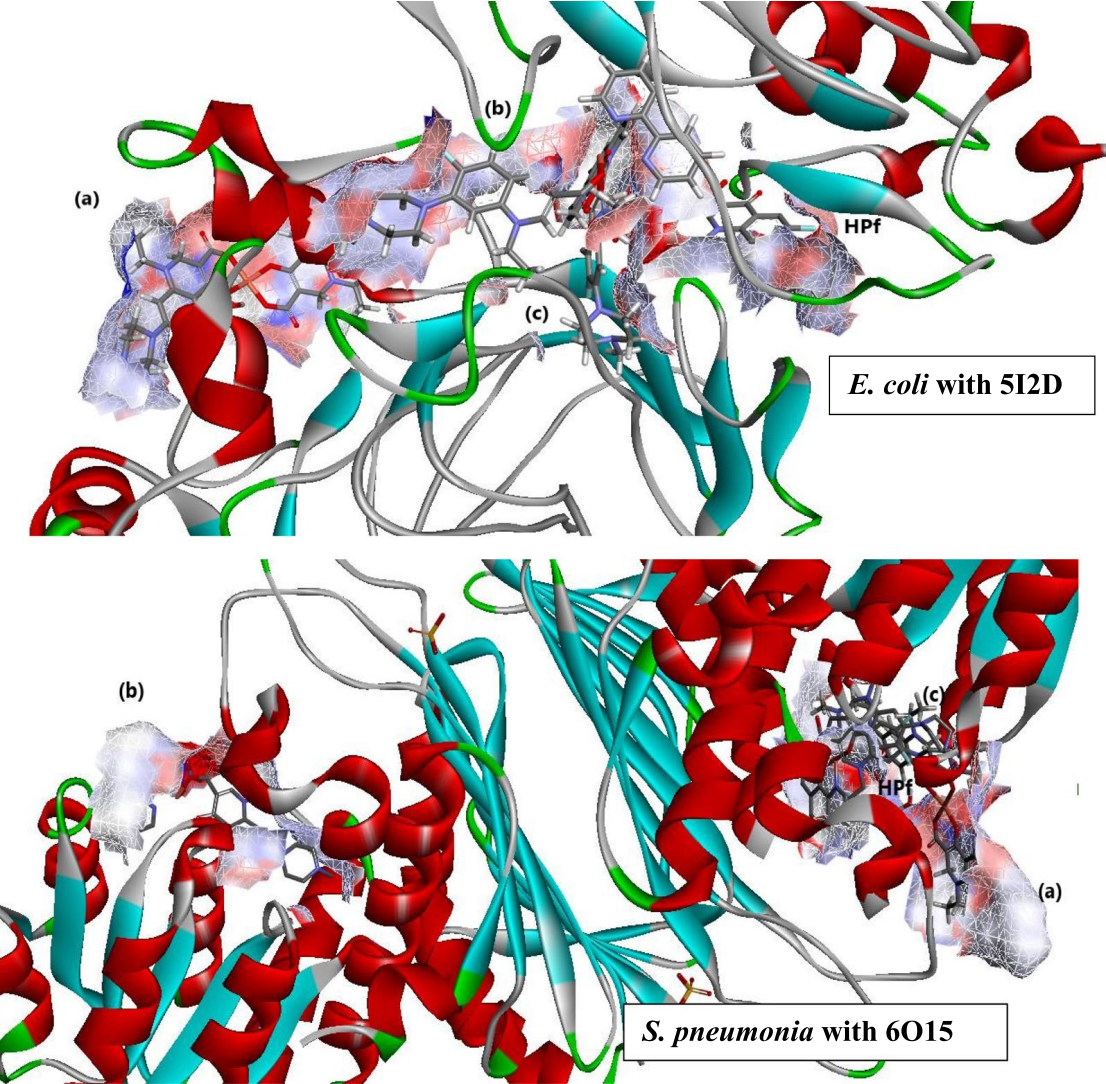
3D-schematic representation of the docked complexes, **a** [Cu(Pf)2(H2O)2].3H2O, **b** [Cu(HPf)(bipy)(NO3)]NO3.2H2O, **c** [Cu(HPf)(phen)(NO3)]NO3.2H2O, compared with HPf, in two protein types with solvated surface accessibility

**Fig. 8:**
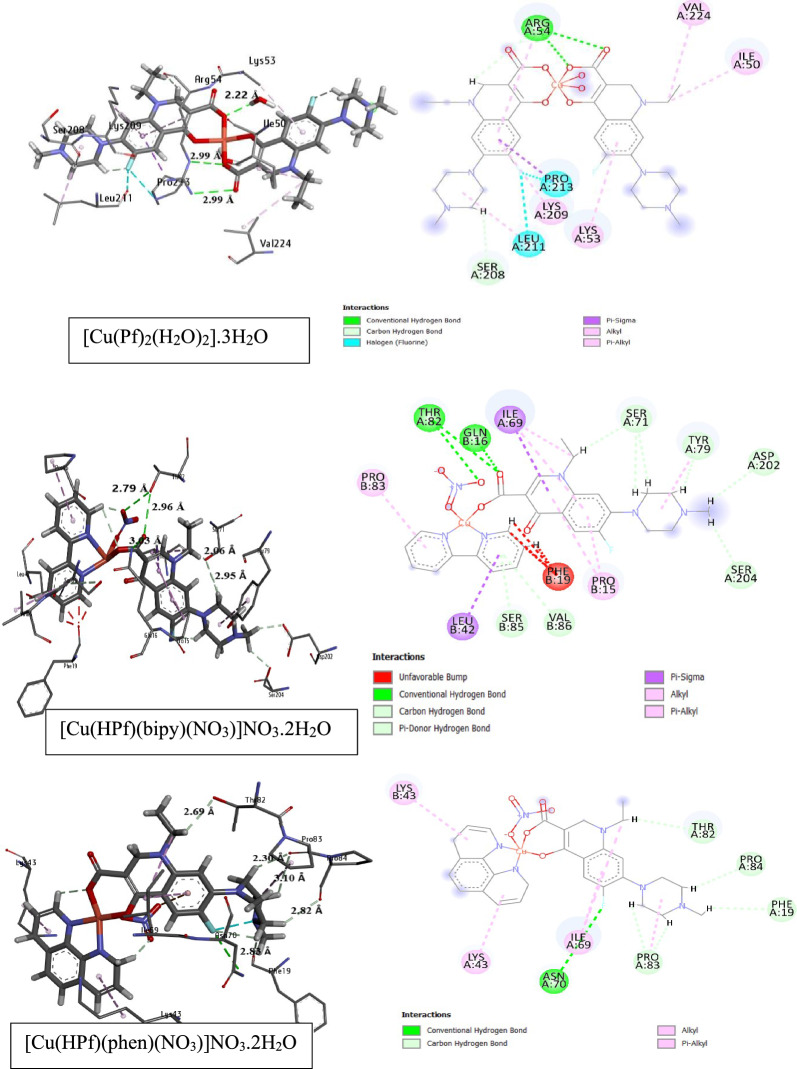
3D and 2D interactions and H-bond distances between *E. coli* amino acids (ID: 5I2D) and the studied complexes

To investigate the bioactive mode, molecular docking was applied to DFT-optimized pefloxacin and its copper complexes, and the docking pocket was downloaded as a PDB file with two protein codes according to the microbial organism (*E. coli and S. pneumoniae).* Pefloxacin is well known as a powerful bioactive agent in the field of pharmaceutical drugs based on its higher functional activity owing to the best score achievement [80–83]. Herein, computational docking will investigate the protein binding modes of pefloxacin copper complexes. Figure 7 represents the 3D docking analysis of the studied complexes with 5I2D and 6O15 protein targets involving solvent accessible surfaces. Additional file 1: Tables S16 and S17 include the evaluated energies and discuss the comparable validity of the studied complexes to bind with different amino acids of *E. coli* protein ID: 5I2D and *S. pneumonia* (ID: 6O15) 6O15. According to the experimental data illustrating the antimicrobial activity, it was found that [Cu(Pf)2(H2O)2]0.3H2O had a better antimicrobial activity, and a molecular docking screen confirmed this result since [Cu(Pf)2(H2O)2]0.3H2O has a higher fitness (TBE = − 107.7 kcal/mol) than the other two complexes. The higher fitness comes from binding with 21 amino acids in the protein pocket, where the total fitness (TBE) of pefloxacin with protein is − 87.3 kcal/mol. Figure 8 shows the number of amino acids interacting with the optimized complexes in different interaction types, such hydrogen bonding (conventional and carbon), alkyl and π-stacking interactions.

**Fig. 9:**
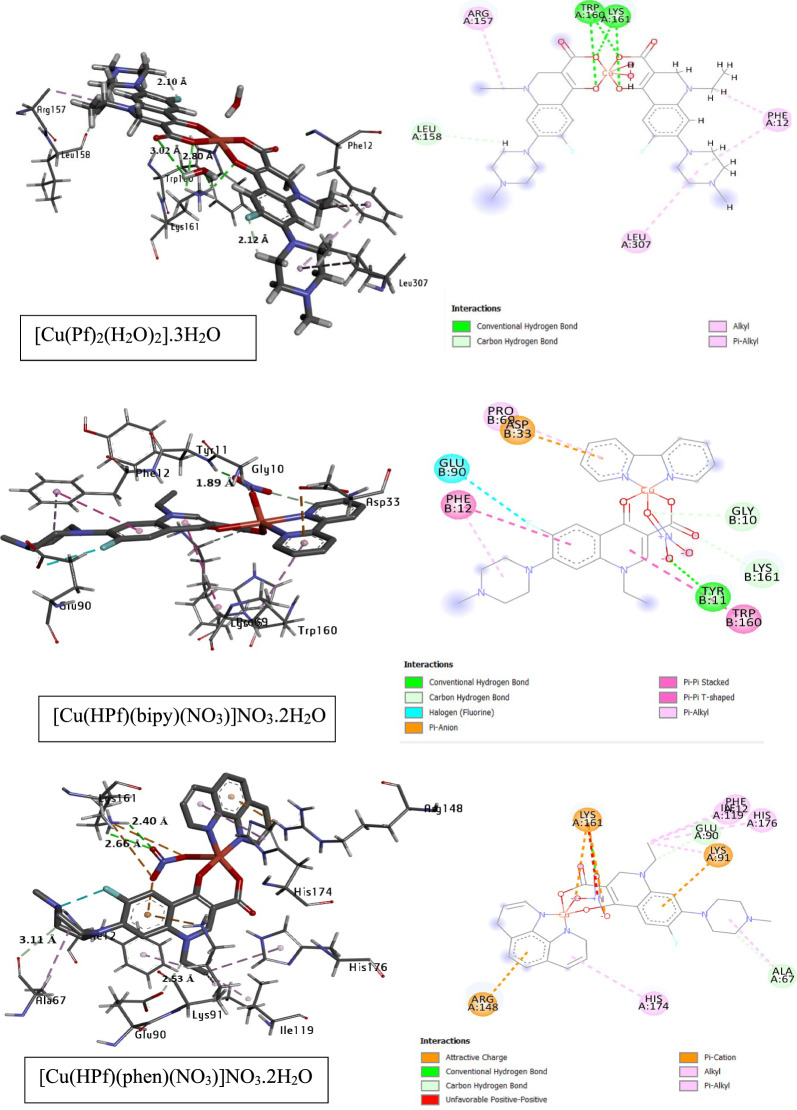
3D and 2D interactions and H-bond distances between *S. pneumoniae* amino acids (ID: 6O15) and the studied complexes

To investigate the docking behavior on other bacterial target types, the protein crystal structure of *S. pneumoniae* with the 6O15 code was chosen. Additional file 1: Table S18 shows the fitness values of the three studied complexes compared with pefloxacin, where [Cu(HPf)(bipy)(NO_3_)]NO_3_.2H_2_O has a fitness score (TBE = − 116.150 kcal/mol) during the docking process. This result does not agree with the experimental data where [Cu(Pf)_2_(H2O)_2_]0.3H_2_O experimentally has the best result, which may be attributed to the mode of docking where the active functional groups attract different numbers of amino acids at the same time as the energy affected by these interactions. There are other intermolecular interactions, such as H-bonding and vdW interactions. Figure 9 demonstrates the labeled interacting amino acids of the 6O15 crystal structure with the studied copper complexes in addition to the presence of the H-bond distance between the interacting parts. Recently, a spectral range of fluoroquinolones (including pefloxacin derivative) were studied against docking performance. G Venkateswara rao *et al* [84] investigated that this class behave as a good inhibiting systems and powerful antibacterial ability towards several bacterial target protein-microbes.

## Conclusion

In summary, three solid copper(II) complexes of pefloxacin (HPf) were synthesized in the absence and presence of two nitrogen donor heterocyclic ligands. The structure of the complexes was investigated by analytical, spectroscopic (FTIR, UV–Vis, EPR, and mass spectrometry), and thermal (TGA, DTG and DTA) techniques. Spectral data suggested the formation of one octahedral binary copper complex and distorted square pyramidal geometry for two ternary copper complexes. Additionally, the pefloxacin –Cu [2]^+^ complex was used as a competitive turn-on fluorescence probe for the detection of amino acid.

A computational study was performed in a gas state, and quantum and reactivity parameters were calculated. Electrophilic and nucleophilic behavior were described using MEP, where the center of copper-pefloxacin in the octahedral binary complex is electron-rich, while it is electron poor in the other two complexes. Pefloxacin and its Cu(II) complexes were in vitro screened against *B.subtilis, S. pneumoniae*, *E. coli*, *P. aeruginosa, C. albicans,* and *A. fumigatus*. The octahedral binary copper complex demonstrated great effectiveness relative to the ternary complexes; thus, it can be considered a powerful antimicrobial broad-spectrum drug that may be able to perform some microbial resistance. Docking simulation was performed with the crystal structure of *E. coli* and *S. pneumoniae* receptors using 5I2D and 6O15 codes, and the interactions occurring in the protein–ligand complexes were predicted. Noncovalent interaction analysis confirmed the presence of different types of interactions in which hydrogen bond formation was the most important interaction.

## Supplementary Information

**Additional file 1: Figure S1: **Mass spectrum of pefloxacin mesylate dehydrate. **Figure S2:** Mass spectrum of [Cu22].3H2O. **Figure S3: **Mass spectrum of [Cu]NO_3_.2H_2_O. **Figure S4: **Mass spectrum of [Cu]NO_3_.2H_2_O. **Figure S5: **Fragmentation pattern of pefloxacin mesylate dehydrate. **Figure S6: **Fragmentation pattern of [Cu_2__2_].3H_2_O. **Figure S7: **Fragmentation pattern of [Cu]NO_3_.2H_2_O. **Figure S8: **Fragmentation pattern of [Cu]NO_3_.2H_2_O. **Figure S9: ** Stern- Volmer plot for the addition of different Cu^2+^ ion concentration to HPf solution at 25 ^o^C and 35 ^o^C. **Figure S10:** Changes of fluorescence intensity after addition of different concentrations of prolinein 0.01 mol L^-1^ phosphate buffer solution: [HPf] = 2.00x10^-7^ mol L^-1^, upon the addition of [Cu^2+^] = 5.00x10^-3^ mol L^-1^, upon the addition of [Cu^2+^] = 5.00x10^-3^ mol L^-1^ and [pro] = 1.00x10^-2^ mol L^-1^, upon the addition of [Cu^2+^] = 5.00x10^-3^ mol L^-1^ and [pro] = 2.00x10^-2^ mol L^-1^**,** upon the addition of [Cu^2+^] = 5.00x10^-3^ mol L^-1^ and [pro] = 3.00x10^-2^ mol L^-1^, upon the addition of [Cu^2+^] = 5.00x10^-3^ mol L^-1^ and [pro] = 4.00x10^-2^ mol L^-1^, upon the addition of [Cu^2+^] = 5.00x10^-3^ mol L^-1^ and [pro] = 4.50x10^-2^ mol L^-1^. **Figure S11:** Changes of fluorescence intensity after addition of different concentrations of alanine in 0.01 mol L^-1^ phosphate buffer solution: [HPf] = 2.00x10^-7^ mol L^-1^**,** upon adding [Cu^2+^] = 5.00x10^-3^ mol L^-1^, upon adding [Cu^2+^] = 5.00x10^-3^ mol L^-1^ and [alanine] = 6.00x10^-2^ mol L^-1^, upon adding [Cu^2+^] = 5.00x10^-3^ mol L^-1^ and [alanine] = 9.00x10^-2^ mol L^-1^**,** upon adding [Cu^2+^] = 5.00x10^-3^ mol L^-1^ and [alanine] = 1.30x10^-1^ mol L^-1^. **Figure S12:** Relative flourescence intensity changes for Cu–pefloxacin complex at 435 nm after the addition of 4.50 x 10^-2^ mol L^-1^ of different amino acids to the 0.01 mol L^-1^ phosphate buffer solution. **Figure S13: **Calibration curves of aspartic acid, proline and alanine in pefloxacin solution at λ_max_ 435 nm in 0.01 mol L^-1^ phosphate buffer solutionat 298 K. **Table S14: **Kinetic parameters of pefloxacin and its complexes. **Table S15. **NBO charge on atoms of pefloxacin and its copper complexes using DFT with CAM-B3LYP/ LanL2DZ/6-311G. **Table S16**: Binding energy distribution of different 5I2D amino acids with pefloxacin and its complexes. **Table S17: **Binding energy distribution of different 6O15 amino acids with pefloxacin and its complexes. **Table S18: **Fitness parameters of docked compounds with *E. coli and S. pneumoniae*.

## Data Availability

All data generated or analyzed during this study are included in this manuscript and supplementary materials.
