# Supplementary material for: A perception into binary and ternary copper (II) complexes: synthesis, characterization, DFT modeling, antimicrobial activity, protein binding screen, and amino acid interaction
Source: BMC Chem. 2023 Jun 14;17(1):55. doi: 10.1186/s13065-023-00962-x (PMC10265897; doi:10.1186/s13065-023-00962-x)
Supplement: Supplementary file 1 — Additional file 1: Figure S1: Mass spectrum of pefloxacin mesylate dehydrate. Figure S2: Mass spectrum of [Cu22].3H2O. Figure S3: Mass spectrum of [Cu]NO3.2H2O. Figure S4: Mass spectrum of [Cu]NO3.2H2O. Figure S5: Fragmentation pattern of pefloxacin mesylate dehydrate. Figure S6: Fragmentation pattern of [Cu22].3H2O. Figure S7: Fragmentation pattern of [Cu]NO3.2H2O. Figure S8: Fragmentation pattern of [Cu]NO3.2H2O. Figure S9: Stern- Volmer plot for the addition of different Cu2+ ion concentration to HPf solution at 25 oC and 35 oC. Figure S10: Changes of fluorescence intensity after addition of different concentrations of prolinein 0.01 mol L-1 phosphate buffer solution: [HPf] = 2.00x10^-7^ mol L-1, upon the addition of [Cu2+] = 5.00x10^-3^ mol L-1, upon the addition of [Cu2+] = 5.00x10^-3^ mol L-1 and [pro] = 1.00x10^-2^ mol L-1, upon the addition of [Cu2+] = 5.00x10^-3^ mol L-1 and [pro] = 2.00x10^-2^ mol L-1, upon the addition of [Cu2+] = 5.00x10^-3^ mol L-1 and [pro] = 3.00x10^-2^ mol L-1, upon the addition of [Cu2+] = 5.00x10^-3^ mol L-1 and [pro] = 4.00x10^-2^ mol L-1, upon the addition of [Cu2+] = 5.00x10^-3^ mol L-1 and [pro] = 4.50x10^-2^ mol L-1. Figure S11: Changes of fluorescence intensity after addition of different concentrations of alanine in 0.01 mol L-1 phosphate buffer solution: [HPf] = 2.00x10^-7^ mol L-1, upon adding [Cu2+] = 5.00x10^-3^ mol L-1, upon adding [Cu2+] = 5.00x10^-3^ mol L-1 and [alanine] = 6.00x10^-2^ mol L-1, upon adding [Cu2+] = 5.00x10^-3^ mol L-1 and [alanine] = 9.00x10^-2^ mol L-1, upon adding [Cu2+] = 5.00x10^-3^ mol L-1 and [alanine] = 1.30x10^-1^ mol L-1. Figure S12: Relative flourescence intensity changes for Cu–pefloxacin complex at 435 nm after the addition of 4.50 x 10^-2^ mol L-1 of different amino acids to the 0.01 mol L-1 phosphate buffer solution. Figure S13: Calibration curves of aspartic acid, proline and alanine in pefloxacin solution at λmax 435 nm in 0.01 mol L-1 phosphate buffer solutionat 298 K. Table S14: Kinetic parameters of pefloxacin and its compl [file 13065_2023_962_MOESM1_ESM.docx]

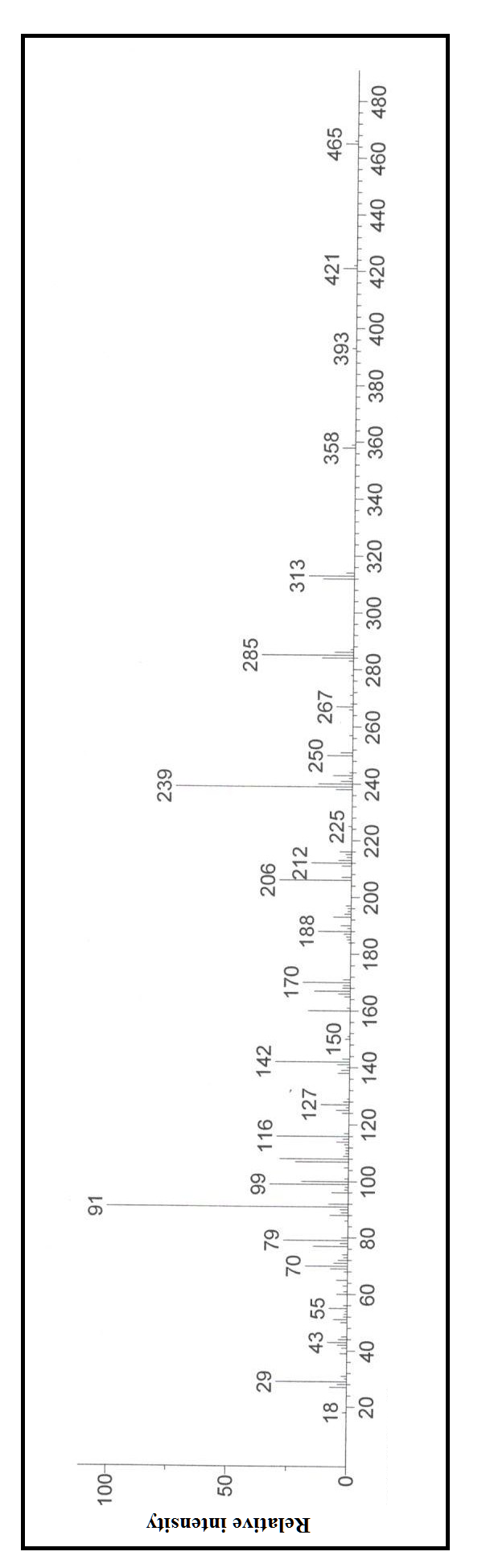


**S1:** Mass spectrum of pefloxacin mesylate dihydrate


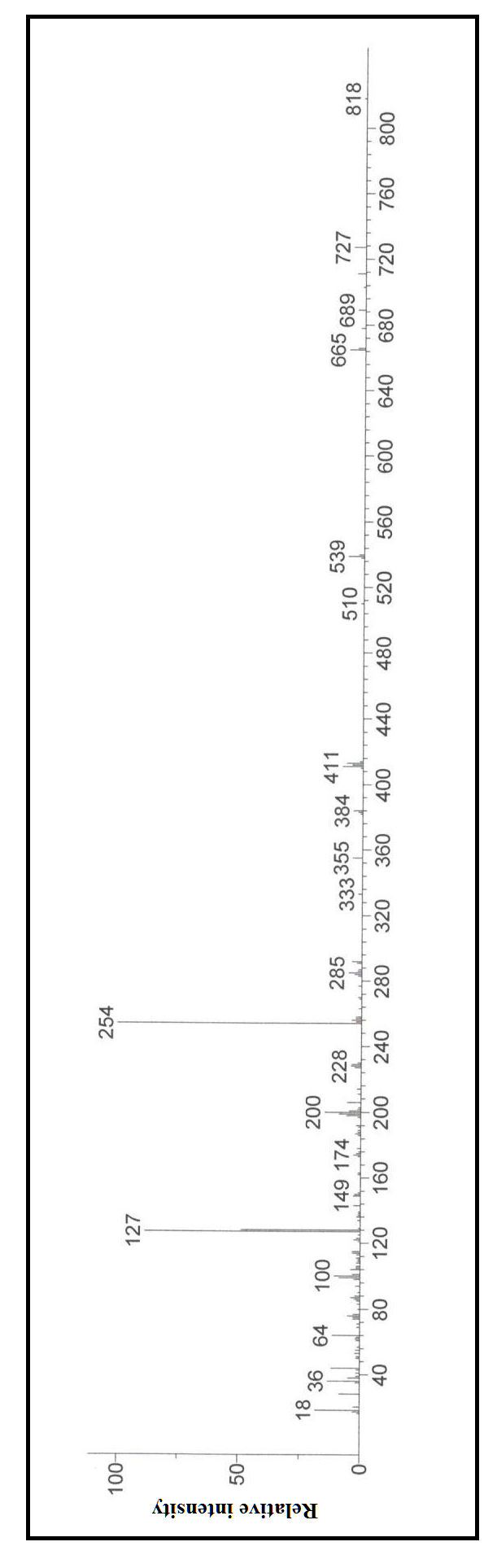


**S2:** Mass spectrum of [Cu(Pf)2(H2O)2].3H2O


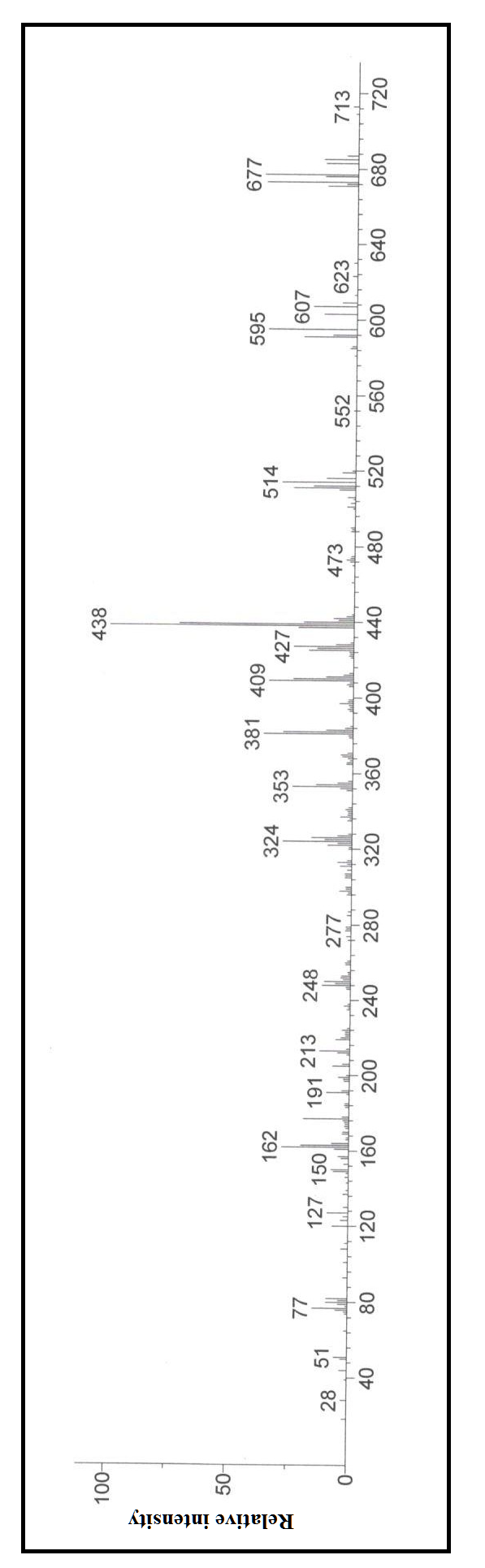


**S3:** Mass spectrum of [Cu(HPf)(bipy)(NO_3_)]NO_3_.2H_2_O


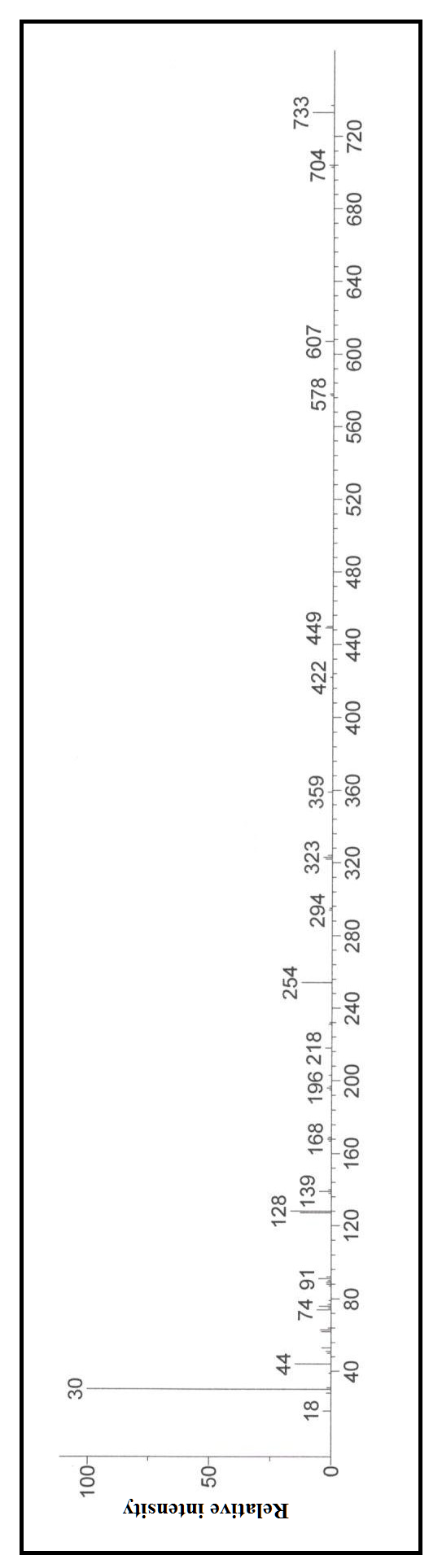


**S4:** Mass spectrum of [Cu(HPf)(phen)(NO_3_)]NO_3_.2H_2_O

**S5:** Fragmentation pattern of pefloxacin mesylate dihydrate

**S6:** Fragmentation pattern of [Cu(Pf)_2_(H_2_O)_2_].3H_2_O

**S7:** Fragmentation pattern of [Cu(HPf)(bipy)(NO_3_)]NO_3_.2H_2_O

**S8:** Fragmentation pattern of [Cu(HPf)(phen)(NO_3_)]NO_3_.2H_2_O

**S9:**  Stern- Volmer plot for the addition of different Cu^2+^ ion concentration to HPf solution at 25 ^o^C and 35 ^o^C


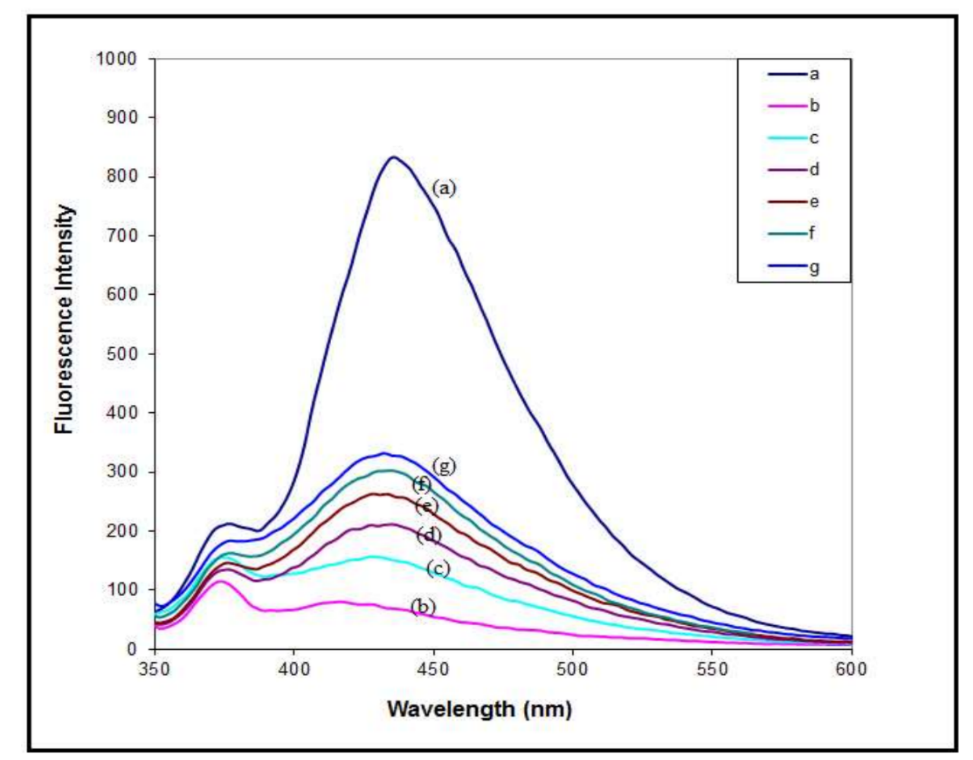


**S10:** Changes of fluorescence intensity after addition of different concentrations of proline (pro) in 0.01 mol L^-1^ phosphate buffer solution (pH 7): **(a)** [HPf] = 2.00x10^-7^ mol L^-1^, **(b)** upon the addition of [Cu^2+^] = 5.00x10^-3^ mol L^-1^, **(c)** upon the addition of [Cu^2+^] = 5.00x10^-3^ mol L^-1^ and [pro] = 1.00x10^-2^ mol L^-1^,  **(d)** upon the addition of [Cu^2+^] = 5.00x10^-3^ mol L^-1^ and [pro] = 2.00x10^-2^ mol L^-1^**, (e)** upon the addition of [Cu^2+^] = 5.00x10^-3^ mol L^-1^ and [pro] = 3.00x10^-2^ mol L^-1^, **(f)** upon the addition of [Cu^2+^] = 5.00x10^-3^ mol L^-1^ and [pro] = 4.00x10^-2^ mol L^-1^, **(g)** upon the addition of [Cu^2+^] = 5.00x10^-3^ mol L^-1^ and [pro] = 4.50x10^-2^ mol L^-1^


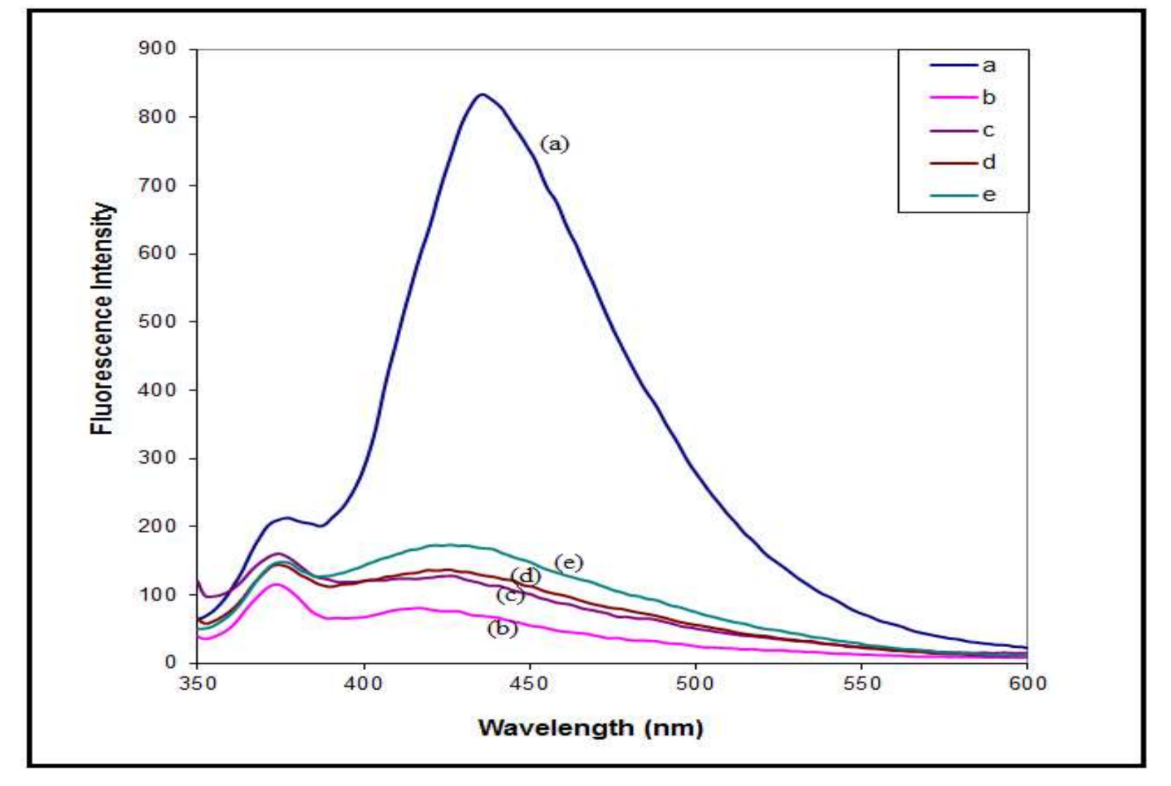


**S11:** Changes of fluorescence intensity after addition of different concentrations of alanine in 0.01 mol L^-1^ phosphate buffer solution (pH7): **(a)** [HPf] = 2.00x10^-7^ mol L^-1^**, (b)** upon adding [Cu^2+^] = 5.00x10^-3^ mol L^-1^, **(c)** upon adding [Cu^2+^] = 5.00x10^-3^ mol L^-1^ and [alanine] = 6.00x10^-2^ mol L^-1^, **(d)** upon adding [Cu^2+^] = 5.00x10^-3^ mol L^-1^ and [alanine] = 9.00x10^-2^ mol L^-1^**, (e)** upon adding [Cu^2+^] = 5.00x10^-3^ mol L^-1^ and [alanine] = 1.30x10^-1^ mol L^-1^


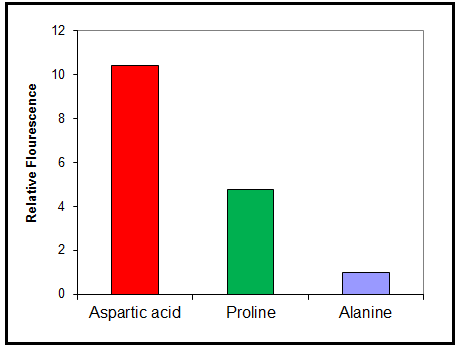


**S12:** Relative flourescence intensity changes for Cu–pefloxacin complex at 435 nm after the addition of 4.50 x 10^-2^ mol L^-1^ of different amino acids to the 0.01 mol L^-1^ phosphate buffer solution


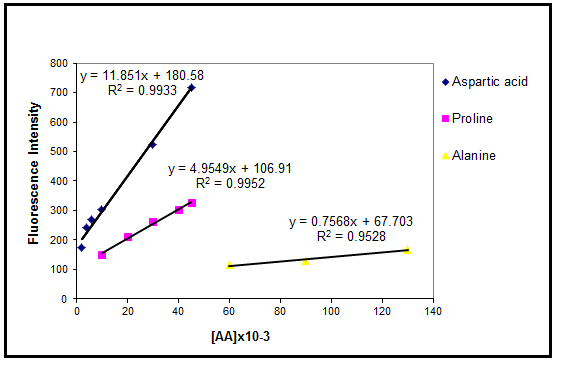


**S13:** Calibration curves of aspartic acid, proline and alanine in pefloxacin solution at λ_max_ 435 nm in 0.01 mol L^-1^ phosphate buffer solution (pH 7) at 298 K

**S14:** Kinetic parameters of pefloxacin and its complexes

| Compound | Peak type | Slope | ∆E | a | B | S | n | α_m_ | T_m_ K) | Z(s^-1^) | ∆S | ∆H | R^2^ |
| --- | --- | --- | --- | --- | --- | --- | --- | --- | --- | --- | --- | --- | --- |
| Pefloxacin mesylate dihydrate | exo  exo  exo | 19.09  115.16  113.40 | \| 75.56 \| \| --- \| \| 126.00 \| \| 111.40 \| | 0.6  0.7  1.1 | 0.7  0.4  1.0 | 0.86  1.75  1.10 | 1.17  1.67  1.32 | 0.603  0.535  0.580 | 333  416  507 | \| 139.8 \| \| --- \| \| 187.7 \| \| 131.4 \| | \| -0.205 \| \| --- \| \| -0.204 \| \| -0.209 \| | \| -68.18 \| \| --- \| \| -84.93 \| \| -105.85 \| | 0.99  0.98  0.99 |
| [Cu(Pf)_2_(H_2_O)_2_].3H_2_O | endo  exo  exo  exo | \| -27.18 \| \| --- \| \| -165.35 \| \| -23.49 \| \| -47.48 \| | \| 225.97 \| \| --- \| \| 1374.72 \| \| 195.32 \| \| 394.71 \| | 0.35  0.2  1.7  1.0 | 0.2  0.4  0.3  0.67 | 1.75  0.50  5.67  1.50 | 1.67  0.89  3.00  1.54 | 0.535  0.653  0.423  0.551 | 348  537  604  649 | \| 461.4 \| \| --- \| \| 2578.7 \| \| 195.8 \| \| 386.5 \| | \| -0.195 \| \| --- \| \| -0.184 \| \| -0.207 \| \| -0.202 \| | \| -67.93 \| \| --- \| \| -99.08 \| \| -124.97 \| \| -131.00 \| | 1  0.99  0.99  1 |
| [Cu(HPf)(bipy)(NO_3_)]NO_3_.2H_2_O | exo  exo | \| -111.09 \| \| --- \| \| -15.10 \| | \| 923.60 \| \| --- \| \| 125.52 \| | 0.2  2.8 | 0.3  1.3 | 0.67  2.15 | 1.03  1.85 | 0.627  0.515 | 563  710 | \| 1322.3 \| \| --- \| \| 103.4 \| | \| -0.190 \| \| --- \| \| -0.214 \| | \| -107.22 \| \| --- \| \| -151.63 \| | 0.98  0.97 |
| [Cu(HPf)(phen)(NO_3_)]NO_3_.2H_2_O | exo  exo | \| -81.23 \| \| --- \| \| -44.49 \| | \| 675.38 \| \| --- \| \| 369.85 \| | 0.3  0.8 | 0.5  1.5 | 0.60  0.53 | 0.98  0.92 | 0.637  0.647 | 546  772 | \| 922.2 \| \| --- \| \| 293.1 \| | \| -0.193 \| \| --- \| \| -0.206 \| | \| -105.48 \| \| --- \| \| -158.72 \| | 0.99  1 |

ΔE: activation energy, S: asymmetry of the peak, n: order of chemical reaction, αm: decomposedsubstance fraction at the moment of maximum development of reaction with (T=T_m_), Z: collision factor (s^-1^), ∆S: entropy in KJ/mol, ∆H: enthalpy in KJ/mol, R^2^: coefficient of determination.

**S15.** NBO charge on atoms of pefloxacin and its copper complexes using DFT with CAM-B3LYP/ LanL2DZ/6-311G(d,p)

| **HPf** | | **[Cu(Pf)_2_(H_2_O)_2_].3H_2_O** | | **[Cu(HPf)(bipy)(NO_3_)]NO_3_.2H_2_O** | | **[Cu(HPf)(phen)(NO_3_)]NO_3_.2H_2_O** | |
| --- | --- | --- | --- | --- | --- | --- | --- |
| **Atom** | **charge** | **atom** | **Charge** | **Atom** | **charge** | **atom** | **charge** |
| C1 | -0.137 | O 1 | -0.974 | C1 | -0.215 | C1 | 0.064 |
| C2 | 0.240 | O 2 | -0.793 | C2 | 0.120 | C2 | -0.071 |
| C3 | -0.068 | O 3 | -0.678 | N1 | -0.571 | C3 | 0.106 |
| C4 | 0.108 | O 4 | -0.686 | C3 | -0.146 | C4 | -0.034 |
| N1 | -0.206 | O 5 | -0.780 | C4 | -0.209 | C5 | 0.108 |
| C5 | -0.103 | C1 | 0.496 | C5 | 0.210 | C6 | -0.070 |
| C6 | 0.197 | C2 | -0.252 | Cu1 | 1.058 | C7 | -0.112 |
| C7 | 0.090 | C3 | -0.134 | O1 | -0.504 | C8 | 0.068 |
| C8 | -0.102 | C4 | 0.216 | N2 | 0.690 | N1 | -0.219 |
| C9 | -0.108 | C5 | 0.490 | O2 | -0.558 | N2 | -0.230 |
| C10 | -0.107 | C6 | -0.130 | O3 | -0.552 | Cu1 | 0.822 |
| C11 | -0.105 | C7 | -0.246 | O4 | -0.780 | O1 | -0.255 |
| N2 | -0.251 | C8 | 0.217 | O5 | -0.717 | O2 | -0.241 |
| C12 | 0.062 | C9 | 0.123 | C6 | -0.246 | O3 | -0.271 |
| C13 | 0.409 | N1 | -0.409 | C7 | 0.145 | N3 | 0.343 |
| C14 | -0.153 | N2 | -0.409 | C8 | -0.111 | O4 | -0.312 |
| N3 | -0.275 | C10 | 0.826 | C9 | 0.491 | C9 | -0.108 |
| C15 | -0.097 | C11 | 0.829 | C10 | 0.223 | C10 | 0.246 |
| C16 | -0.327 | O6 | -0.681 | N3 | -0.395 | C11 | 0.072 |
| O1 | -0.316 | O7 | -0.692 | C11 | -0.274 | C12 | -0.093 |
| O2 | -0.364 | C12 | -0.308 | C12 | -0.186 | C13 | 0.196 |
| O3 | -0.341 | C13 | -0.208 | C13 | 0.399 | C14 | 0.061 |
| C17 | -0.207 | C14 | 0.395 | C14 | -0.193 | C15 | -0.112 |
| F1 | -0.191 | C15 | -0.190 | N4 | -0.513 | N4 | -0.199 |
|  |  | C16 | -0.317 | N5 | -0.490 | N5 | -0.289 |
|  |  | C17 | 0.385 | C15 | -0.219 | N6 | -0.249 |
|  |  | C18 | 0.175 | C16 | -0.218 | C16 | -0.107 |
|  |  | C19 | -0.192 | C17 | -0.227 | C17 | -0.105 |
|  |  | C20 | -0.193 | C18 | -0.211 | C18 | -0.106 |
|  |  | C21 | -0.653 | C19 | -0.403 | C19 | -0.107 |
|  |  | F1 | -0.380 | C20 | -0.657 | C20 | 0.413 |
|  |  | F2 | -0.382 | F1 | -0.380 | O5 | -0.311 |
|  |  | N3 | -0.495 | C21 | -0.143 | O6 | -0.320 |
|  |  | N4 | -0.551 | C22 | 0.127 | C21 | -0.097 |
|  |  | N5 | -0.550 | C23 | -0.215 | C22 | -0.328 |
|  |  | C22 | -0.204 | C24 | -0.222 | C23 | -0.110 |
|  |  | C23 | -0.209 | C25 | 0.220 | C24 | -0.032 |
|  |  | C24 | -0.213 | N6 | -0.578 | C25 | -0.087 |
|  |  | C25 | -0.413 | C26 | 0.827 | C26 | -0.088 |
|  |  | C26 | -0.413 | O6 | -0.642 | C27 | 0.113 |
|  |  | O8 | -1.022 | C27 | 0.179 | C28 | -0.039 |
|  |  | Cu1 | 1.07518 |  |  | F1 | -0.205 |
|  |  | C27 | -0.206 |  |  | C29 | -0.203 |
|  |  | C28 | -0.220 |  |  |  |  |
|  |  | C29 | -0.202 |  |  |  |  |
|  |  | C30 | -0.222 |  |  |  |  |
|  |  | C31 | 0.180 |  |  |  |  |
|  |  | C32 | -0.654 |  |  |  |  |
|  |  | N6 | -0.502 |  |  |  |  |
|  |  | C33 | -0.216 |  |  |  |  |
|  |  | C34 | 0.124 |  |  |  |  |

**S16**: Binding energy distribution of different 5I2D amino acids with pefloxacin and its

complexes

| Compound | Pefloxacin | [Cu(Pf)_2_(H_2_O)_2_].3H_2_O | [Cu(HPf)(bipy)(NO_3_)]NO_3_.2H_2_O | [Cu(HPf)(phen)(NO_3_)]NO_3_.2H_2_O |
| --- | --- | --- | --- | --- |
| TBE (kcal/mol) | -87.300 | -107.700 | -98.500 | -100.600 |
| ARG-54 | 0.000 | -10.521 | 0.000 | 0.000 |
| SER-71 | -3.500 | 0.000 | -3.863 | 0.000 |
| TYR-79 | 0.000 | 0.000 | 0.000 | 0.000 |
| THR-82 | 0.000 | 0.000 | -7.003 | 0.000 |
| GLN-16 | 0.000 | 0.000 | -4.621 | 0.000 |
| SER-85 | -3.058 | 0.000 | 0.000 | 0.000 |
| SER-85 | -2.255 | 0.000 | -5.899 | 0.000 |
| LEU-42 | 0.000 | 0.000 | 0.000 | -5.736 |
| LEU-42 | 0.000 | 0.000 | 0.000 | -5.586 |
| LYS-43 | 0.000 | 0.000 | 0.000 | -6.713 |
| GLU-49 | 0.000 | -4.587 | 0.000 | 0.000 |
| ILE-50 | 0.000 | -8.148 | 0.000 | 0.000 |
| ILE-50 | 0.000 | -8.672 | 0.000 | 0.000 |
| LYS-53 | 0.000 | -5.674 | 0.000 | 0.000 |
| ARG-54 | 0.000 | -11.504 | 0.000 | 0.000 |
| ARG-54 | 0.000 | -12.312 | 0.000 | 0.000 |
| ILE-69 | -4.382 | 0.000 | -7.151 | -10.243 |
| ASN-70 | -2.363 | 0.000 | -1.729 | -7.082 |
| ASN-70 | 0.000 | 0.000 | 0.000 | -6.065 |
| TYR-79 | -4.185 | 0.000 | 3.537 | 0.000 |
| GLY-80 | -6.613 | 0.000 | -3.967 | 0.000 |
| LYS-81 | -4.490 | 0.000 | -2.012 | 0.000 |
| THR-82 | -4.124 | 0.000 | -2.337 | -1.246 |
| THR-82 | -6.308 | 0.000 | -5.042 | -3.862 |
| PRO-83 | -0.600 | 0.000 | -0.357 | -4.604 |
| PRO-84 | 0.000 | 0.000 | 0.000 | -5.102 |
| SER-208 | 0.000 | -6.297 | 0.000 | 0.000 |
| LYS-209 | 0.000 | -4.964 | 0.000 | 0.000 |
| LYS-209 | 0.000 | -5.520 | 0.000 | 0.000 |
| LEU-211 | 0.000 | -4.105 | 0.000 | 0.000 |
| PRO-213 | 0.000 | -8.478 | 0.000 | 0.000 |
| ASP-14 | -2.214 | 0.000 | -7.712 | 0.000 |
| PRO-15 | -4.129 | 0.000 | -7.443 | 0.000 |
| LYS-20 | 0.000 | 0.000 | -5.503 | -0.054 |
| LYS-43 | 0.000 | 0.000 | 0.000 | -5.630 |
| THR-82 | -6.563 | 0.000 | 0.836 | -2.078 |
| PRO-83 | -4.118 | 0.000 | 2.206 | -0.189 |
| PRO-84 | -3.115 | 0.000 | -6.907 | 0.000 |
| SER-85 | -1.889 | 0.000 | -8.03612 | 0.000 |
| SER-85 | -2.597 | 0.000 | -5.529 | -0.186 |
| VAL-86 | 0.000 | 0.000 | -4.251 | 0.000 |

**S17:** Binding energy distribution of different 6O15 amino acids with

pefloxacin and its complexes

| Compound | Pefloxacin | [Cu(Pf)2(H2O)2].3H2O | [Cu(HPf)(bipy)(NO3)]NO3.2H2O | [Cu(HPf)(phen)(NO3)]NO3.2H2O |
| --- | --- | --- | --- | --- |
| TBE | -92.445 | -110. 464 | -116.150 | -115.371 |
| LYS-91 | -3.500 | 0.000 | 0.000 | 0.000 |
| ARG-148 | -3.500 | 0.000 | 0.000 | -2.500 |
| TRP-160 | 0.000 | -6.582 | 0.000 | 0.000 |
| LYS-161 | 0.000 | -7.000 | 0.000 | -3.500 |
| HIS-173 | -2.500 | 0.000 | 0.000 | 0.000 |
| HIS-174 | -3.500 | 0.000 | 0.000 | 0.000 |
| HIS-176 | -5.643 | 0.000 | 0.000 | -3.321 |
| TYR-11 | 0.000 | 0.000 | -8.691 | 0.000 |
| TRP-160 | 0.000 | 0.000 | -6.641 | 0.000 |
| HIS-176 | 0.000 | 0.000 | -2.500 | 0.000 |
| TYR-11 | -1.141 | -9.892 | 0.000 | -3.226 |
| PHE-12 | -1.439 | -4.739 | 0.000 | -3.117 |
| PHE-12 | -6.196 | -12.515 | 0.000 | -14.098 |
| LYS-91 | -5.345 | -1.565 | 0.000 | -9.889 |
| ARG-148 | -2.089 | -0.215 | 0.000 | -6.361 |
| LEU-158 | 0.000 | -4.351 | 0.000 | 0.000 |
| SER-159 | 0.000 | -4.674 | 0.000 | 0.000 |
| SER-159 | 0.000 | -4.819 | 0.000 | 0.000 |
| TRP-160 | -9.093 | -13.868 | 0.000 | -6.482 |
| LYS-161 | -5.702 | -2.973 | 0.000 | -5.491 |
| HIS-173 | -9.001 | -1.157 | 0.000 | -9.384 |
| HIS-174 | -6.192 | -0.254 | 0.000 | -7.199 |
| HIS-176 | -1.570 | 0.931 | 0.000 | -7.260 |
| GLY-10 | 0.000 | 0.000 | -4.367 | 0.000 |
| TYR-11 | 0.000 | 0.000 | -6.894 | 0.000 |
| TYR-11 | 0.000 | 0.000 | -9.316 | 0.000 |
| PHE-12 | 0.000 | 0.000 | -6.406 | 0.000 |
| PHE-12 | 0.000 | 0.000 | -10.035 | 0.000 |
| ASP-33 | 0.000 | 0.000 | -6.480 | 0.000 |
| ASN-36 | 0.000 | 0.000 | -4.414 | 0.000 |
| GLU-90 | 0.000 | 0.000 | -5.134 | 0.000 |
| TRP-160 | 0.000 | 0.000 | -8.927 | 0.000 |

**S18:** Fitness parameters of docked compounds with *E. coli* (5I2D) *and S. pneumoniae*

(6O15)

| Compound | | *E.Coli* | | | *S. pneumoniae* | | |
| --- | --- | --- | --- | --- | --- | --- | --- |
|  | | Energy | vdW | HBond | Energy | vdW | HBond |
| [Cu(HPf)(bipy)(NO3)]NO3.2H2O | -98.475 | -76.346 | -22.129 | -116.150 | -96.890 | -19.332 |  |
| [Cu(HPf)(phen)(NO3)]NO3.2H2O | -100.571 | -100.571 | 0.000 | -115.371 | -106.833 | -9.321 |  |
| [Cu(Pf)2(H2O)2].3H2O | -107.651 | -95.366 | -12.286 | -110.464 | -96.883 | -13.582 |  |
| Pefloxacin | -87.257 | -78.444 | -8.813 | -92.445 | -67.267 | -18.643 |  |
